# Enhanced snoMEN Vectors Facilitate Establishment of GFP–HIF-1α Protein Replacement Human Cell Lines

**DOI:** 10.1371/journal.pone.0154759

**Published:** 2016-04-29

**Authors:** Motoharu Ono, Kayo Yamada, Dalila Bensaddek, Vackar Afzal, John Biddlestone, Brian Ortmann, Sharon Mudie, Vincent Boivin, Michelle S. Scott, Sonia Rocha, Angus I. Lamond

**Affiliations:** 1 Centre for Gene Regulation and Expression, School of Life Sciences, University of Dundee, Dundee, United Kingdom; 2 Department of Biochemistry and RNA Group, Faculty of Medicine and Health Sciences, University of Sherbrooke, Sherbrooke, Canada; Univ. of Edinburgh, UNITED KINGDOM

## Abstract

The snoMEN (snoRNA Modulator of gene ExpressioN) vector technology was developed from a human box C/D snoRNA, HBII-180C, which contains an internal sequence that can be manipulated to make it complementary to RNA targets, allowing knock-down of targeted genes. Here we have screened additional human nucleolar snoRNAs and assessed their application for gene specific knock-downs to improve the efficiency of snoMEN vectors. We identify and characterise a new snoMEN vector, termed 47snoMEN, that is derived from box C/D snoRNA U47, demonstrating its use for knock-down of both endogenous cellular proteins and G/YFP-fusion proteins. Using multiplex 47snoMEM vectors that co-express multiple 47snoMEN in a single transcript, each of which can target different sites in the same mRNA, we document >3-fold increase in knock-down efficiency when compared with the original HBII-180C based snoMEN. The multiplex 47snoMEM vector allowed the construction of human protein replacement cell lines with improved efficiency, including the establishment of novel GFP–HIF-1α replacement cells. Quantitative mass spectrometry analysis confirmed the enhanced efficiency and specificity of protein replacement using the 47snoMEN-PR vectors. The 47snoMEN vectors expand the potential applications for snoMEN technology in gene expression studies, target validation and gene therapy.

## Introduction

**Fig 1:**
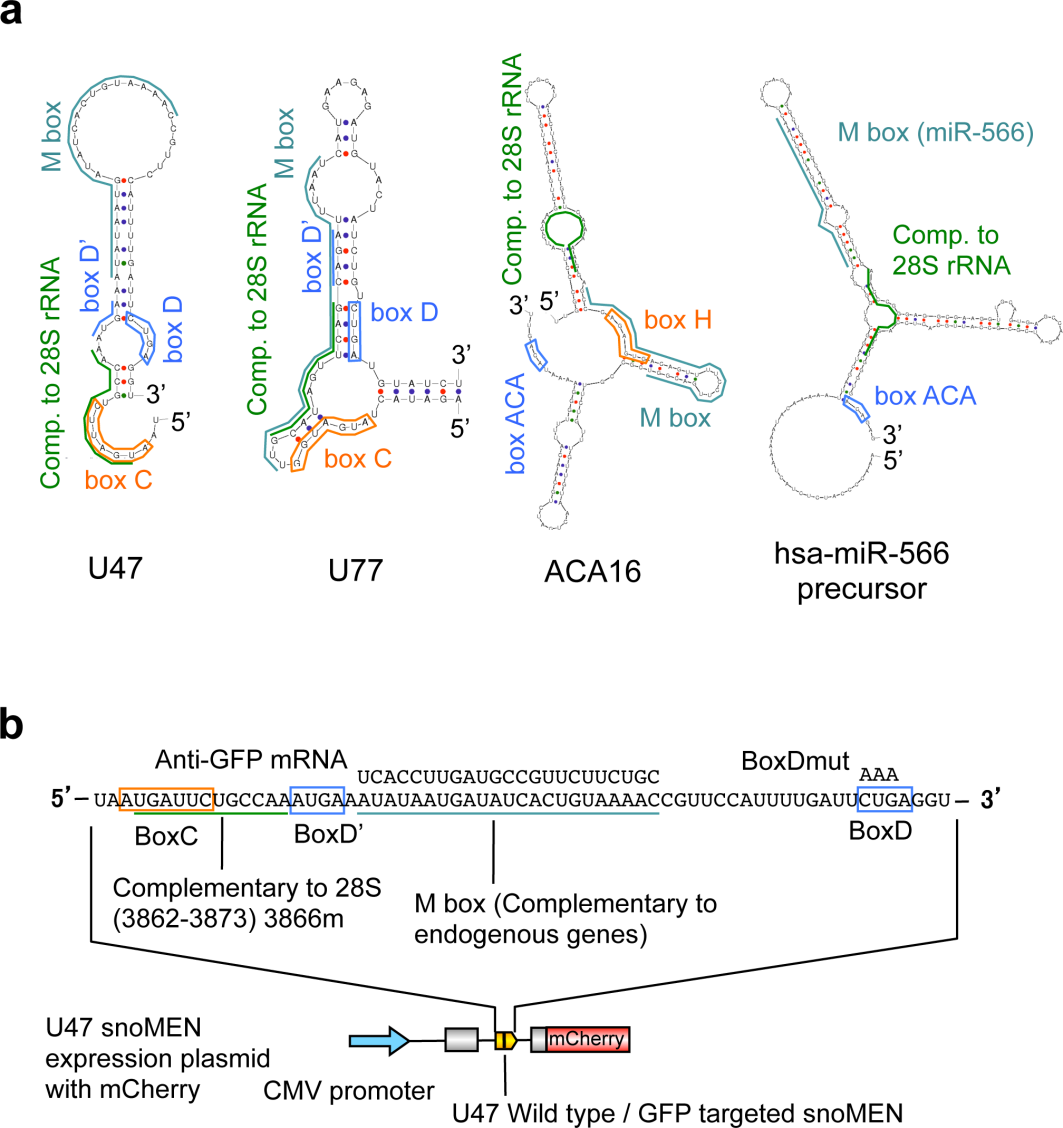
Structures of newly characterised snoMEN vectors. (**a**) Predicted secondary structure of the candidate snoRNAs used for snoMEN vectors. The characteristic features of members of the box C/D and box H/ACA snoRNAs are shown. Conserved box C & D and H & ACA motifs are indicated by orange and blue boxes, respectively. The positions of rRNA complementary sequences are indicated by a green bar. The positions of the M box regions that can be altered to modulate target gene expression (snoMEN vector) are indicated by a cyan bar. (**b**) The vector structure used for targeted suppression of GFP fusion proteins. The sequence in wild type U47 snoRNA that is complementary to pre-mRNAs was changed from 5’-AUAUAAUGAUAUCACUGUAAAAC-3’ to 5’-UCACCUUGAUGCCGUUCUUCUGC-3’ (U47 snoMEN). The resulting U47 snoMEN was subcloned into the 5’ region of the vector with mCherry fluorescent protein cDNA. Diagram shows complementary regions of U47 snoMEN targeting the GFP cDNA sequence (Anti-GFP mRNA). A mutant U47 snoMEN expression plasmid with the box D core motif mutatated was also constructed, shown as BoxDmut on the sequence. The motif labels are the same as in (**a**).

Small nucleolar RNAs (snoRNAs) are a class of conserved RNAs first identified as guides for site specific post-transcriptional modifications in ribosomal RNA (rRNA) [1–4]. The majority of snoRNAs are processed from introns and carry out their function in complex with specific protein interactors, forming ribonucleoprotein complexes, which are referred to as small nucleolar ribonucleoproteins (snoRNPs). Two main groups of snoRNAs have been described. Box C/D snoRNAs form functional complexes *in vivo* with snoRNA associated proteins, such as NOP56, NOP58, 15.5K and the highly conserved protein fibrillarin, which is responsible for rRNA 2′-O-ribose methylation. Human box C/D snoRNA molecules are typically 70–120 nucleotides in length and are mainly encoded in the introns of protein-coding genes. They can be excised from introns through at least two distinct pathways, then further processed and assembled with conserved proteins, including the 2’-O-methyl transferase fibrillarin [4, 5]. Box C/D snoRNAs are characterized by the presence of two, short, conserved motifs, i.e., the C box (UGAUGA) and the D box (CUGA), found near the 5’ and 3’ ends of the molecule, respectively (for example, see **Fig 1A**). The guide sequence with complementarity to the target is located immediately 5’ to the stem II & box D region.

The box H/ACA snoRNAs, which bind the four conserved core proteins DKC1 (dyskerin), GAR1, NHP2 and NOP10, catalyse pseudouridylation at specific target sites on rRNA [2, 3]. Human box H/ACA snoRNAs consist of two hairpins and two short single-stranded regions, which contain the H box (ANANNA) and the ACA box. The latter is always located 3 nucleotides 5' of the 3' end of the snoRNA (for example, see **Fig 1A**). The hairpins contain bulges, or recognition loops, that form complex pseudo-knots with the target RNA, where the target uridine is the first unpaired base. The position of the substrate uridine always resides 14–16 nucleotides upstream of either the H box, or of the ACA box. Some box H/ACA snoRNAs can guide the modification of two uridines, sometimes in two different rRNAs.

Many box C/D and box H/ACA snoRNAs and a subset of their target sites in rRNA are conserved from yeast through to mammalian cells. However, numerous orphan box C/D and box H/ACA snoRNAs have also been identified that do not encode a region of complementarity to rRNA and whose functions remain unknown [4, 6].

We have previously characterised human nucleolar snoRNAs that co-purify with nucleoli isolated from HeLa cells. This led us to identify a novel group of related fibrillarin-associated box C/D snoRNAs that allowed the development of a new vector-based technology for the targeted knock-down of expression of one or more proteins in mammalian cells, termed snoMEN (snoRNA Modulator of Gene Expression) [7, 8]. The snoMEN vectors were developed from modified versions of the human box C/D small nucleolar RNA (snoRNA) HBII-180C (also known as SNORD88C). To knock-down the expression levels of specific gene targets, a short internal snoRNA region, termed the M box, is manipulated to make it complementary to the selected target RNA sequence. Briefly, specific features of the snoMEN vector technology, in comparison with other knock-down systems, include a) snoMEN target nuclear RNAs, e.g. pre-mRNAs and non-coding RNAs [8, 9], b) snoMEN RNAs are transcribed from RNA polymerase II promoters, rather than the RNA polymerase III promoters used in shRNA plasmids, c) multiple snoMEN RNAs can be incorporated within a single transcript and expressed under the regulation of a single promoter [7].

The snoMEN vectors can be used for a wide variety of gene-regulation studies, including knock-down and/or knock-in analysis. For example, snoMEN vectors have been shown to facilitate the establishment of human protein replacement stable cell lines, targeting genes encoding proteins that are problematic to target using shRNA vectors [9]. Knock-down strategies will in practice result in incomplete removal of the targeted protein, with only either chromosomal deletion, or mutation, of the cognate gene guaranteeing complete removal of the protein. Nonetheless, analysis of snoMEN protein replacement stable cell lines showed that even although the protein replacement was partial, it was still sufficient to allow the establishment of stable cell lines for proteins that are toxic when exogenous fusion proteins are simply overexpressed in the presence of unaltered levels of the endogenous factors [9].

In this study, we screen additional human snoRNAs for use as snoMEN vectors and report the identification of novel snoMEN that can deliver enhanced efficiency of knock down and protein replacement. We show these enhanced snoMEN vectors allow the establishment of new human protein replacement stable cell lines, including replacement of the endogenous HIF-1α protein with GFP–HIF-1α, which previously was refractive to knock-in using alternative technological approaches.

## Results

### snoRNA candidates of snoMEN knock-down vector

First, to identify novel snoMEN candidates, we investigated how many human snoRNAs display regions of complementarity to sequences in pre-mRNA, that are at least as long as those present in HBII-180C [7], using the human genome database [10]. This *in silico* analysis identified that 374 of 377 human snoRNAs have a potential M box (messenger RNA complementary box) sequence (**S1 Data**). Next, we selected 8 candidate snoRNAs from this list, prioritising snoRNAs identified in a human nucleolar cDNA library that show high expression levels [7]. The selected snoRNAs analysed were HBII-52, HBII-85, HBII-239, U47, U77, ACA5, ACA16 and hsa-miR-566 precursor. We carried out preliminary experiments to test whether the above set of 8 snoRNAs will show any knock-down of a targeted GFP reporter protein when their endogenous M-box sequence is replaced with a sequence complementary to GFP mRNA. Transient transfection experiments indicated that 4 of these 8 snoRNAs, i.e. U47, U77, ACA16 and hsa-miR-566 precursor, showed some degree of knock-down of GFP fluorescence levels, relative either to mock transfected control cells, or to cells transiently transfected with control vectors not targeted to GFP (data not shown). Therefore, we concentrated follow up analysis on these 4 snoRNAs (**Fig 1A** and **Fig A** in **S1 File**).

The U47/U77 and ACA16 are well-characterised box C/D and box H/ACA snoRNAs, respectively [11–15]. The hsa-miR-566 precursor was annotated as a precursor of microRNA 566 (miR-566) from deep sequencing studies [16]. However, our nucleolar cDNA library identified that there are miR-566 precursor molecules in purified human nucleolar preparations and we have validated this in multiple clones (data not shown). Analysis of the miR-566 precursor RNA sequence shows a predicted secondary structure resembling a canonical box H/ACA snoRNA (**Fig 1A**). Furthermore, experimentally we find this RNA is enriched in purified nucleoli (**Fig B** in **S1 File**). Therefore, we propose that miR-566 is a snoRNA derived RNA (sdRNA) and that the miR-566 precursor is actually a box H/ACA snoRNA-like molecule.

**Fig 2:**
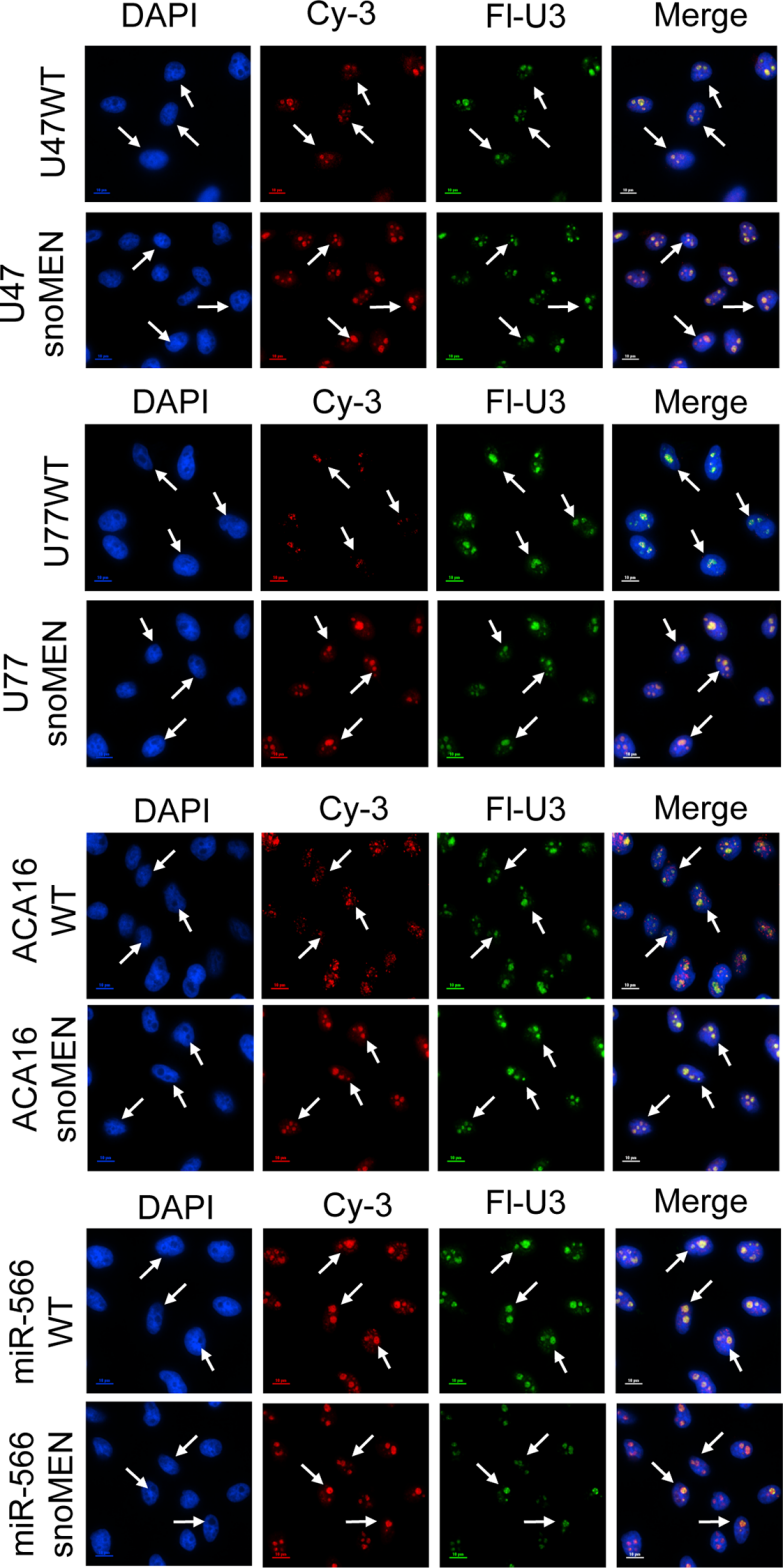
Expression and localization of snoMEN snoRNAs. Fluorescence *In Situ* Hybridisation shows nuclear localization and also nucleolar accumulation for wild type snoRNAs, i.e. U47, U77, ACA16, miR566, and snoMEN, i.e. U47snoMEN, U77snoMEN, ACA16snoMEN, miR566snoMEN (Cy3, Red). U3snoRNA specific probe was also co-hybridised as nucleolar marker (Fl, Green). DNA is stained by DAPI (Blue). Scale Bar is 10 μm.

To evaluate these 4 snoRNAs in more detail for use as a snoRNA modulator of gene expression (snoMEN) vector, we next examined their subcellular localization. Each snoRNA was transiently expressed with its endogenous M box region replaced by a sequence complementary to G/YFP mRNA and subcloned in a plasmid vector upstream (5’) of mCherry fluorescent protein cDNA, which provides a transfection marker (**Fig 1B** and **Figs C-E** in **S1 File**). Fluorescent *in situ* hybridisation (FISH) analysis was performed on HeLa cells, using fluorescent labelled snoMEN-specific probes, following transfection of the HeLa cells with each of these vector constructs. In each case this showed a consistent nuclear localisation pattern for the exogenously expressed, modified snoRNA, with prominent accumulation in nucleoli. A similar nucleolar labelling pattern was also seen upon expression of wild type versions of each snoRNA, i.e. not complementary to G/YFP mRNA (**Fig 2** WT).

### snoMEN RNAs bind core snoRNP proteins

**Fig 3:**
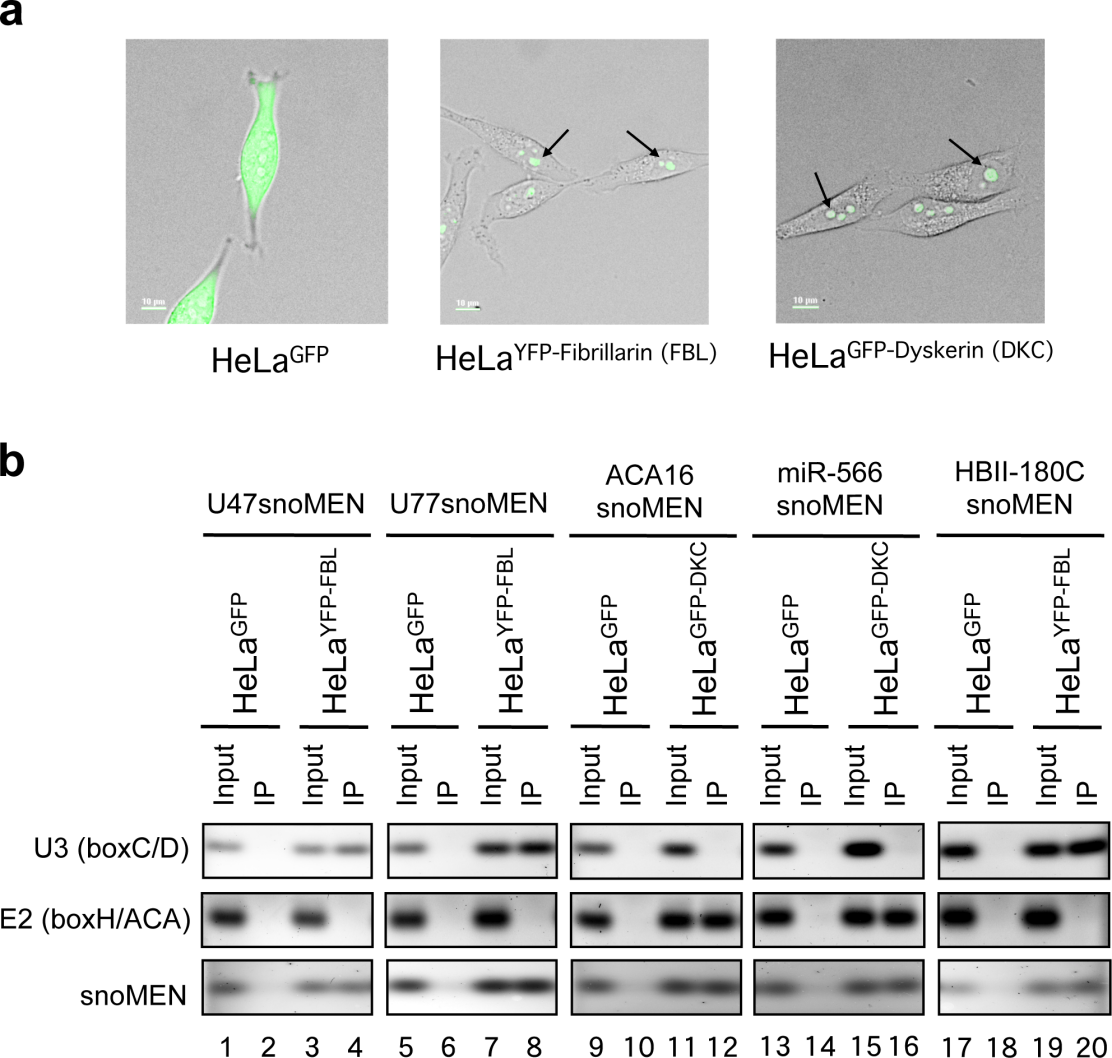
Co-immunoprecipitation of snoMEN with snoRNP core proteins. (**a**) Live cell images of HeLa^GFP^, HeLa^YFP-Fibrillarin (FBL)^, and HeLa^GFP-Dyskerin (DKC)^ stable cell lines that stably express free GFP, YFP-Fibrillarin and GFP-Dyskerin, respectively. (**b**) Extracts were prepared from HeLa cell lines stably expressing either YFP-Fibrillarin, or free GFP, for box C/D snoRNAs (U47, U77, HBII-180C) and either GFP-Dyskerin, or free GFP, for box H/ACA snoRNAs (ACA16 and miR566), after transfection of snoMEN expression vectors, as indicated at the top (U47snoMEN, U77snoMEN, ACA16snoMEN, miR-566snoMEN, and HBII-180CsnoMEN). Extracts were immunoprecipitated using a monoclonal anti-GFP antibody conjugated to beads (GFP-TRAP_A), with the specificity confirmed by western blotting [20, 33]. Quantitative RT-PCR was used to detect co-precipitated U47 snoMEN, U77 snoMEN, ACA16 snoMEN, miR566 snoMEN and HBII-180C snoMEN snoRNAs (snoMEN), using U3 (box C/D) and E2 (box H/ACA) snoRNAs as a positive and negative control, respectively, for fibrillarin/dyskerin-associated RNAs. Equal amounts of material were loaded on the IP and Input lanes.

We next investigated whether the snoMEN RNAs bind specifically to either fibrillarin (FBL), a core component of box C/D snoRNPs, or to Dyskerin (DKC), a core component of box H/ACA snoRNPs. HeLa cells expressing either YFP-Fibrillarin, or GFP, as a control, were transfected with either U47snoMEN, U77snoMEN, or the original snoMEN RNA HBII-180C [7] and nuclei isolated from the transfected HeLa cells. Similar experiments were performed transfecting HeLa cells with ACA16snoMEN and miR-566snoMEN, except GFP-Dyskerin expressing HeLa cells were used instead of the HeLa YFP-Fibrillarin stable cell line (**Fig 3A**). Immunoprecipitations were carried out from the resulting nuclear lysates, using an antibody specific for the G/YFP fluorescent proteins (see Methods). The RNA was isolated from these samples and analysed by qRT-PCR (see Methods). As shown in **Fig 3B** (lanes 4, 8 and 20), U47snoMEN, U77snoMEN and HBII-180C snoMEN co-purify specifically with fibrillarin, as does the positive control U3 snoRNA. In contrast, the box H/ACA snoRNA E2 is not pulled down with YFP-fibrillarin (**Fig 3B** lanes 4, 8 and 20). Similar results were observed for ACA16snoMEN and miR-566snoMEN, where the snoMEN co-purify specifically with dyskerin (**Fig 3B** lanes 12 and 16), as does the E2 box H/ACA snoRNA. In contrast, the control U3 box C/D snoRNA is not pulled down with GFP-dyskerin. The combination of these approaches, i.e., nucleolar cDNA cloning, secondary structure analysis, nucleolar localisation, fibrillarin and dyskerin binding experiments, demonstrate that all of these 4 modified human snoRNAs behave similarly to the previously analysed HBII-180C snoMEN [7].

### Modulated expression of targeted genes

**Fig 4:**
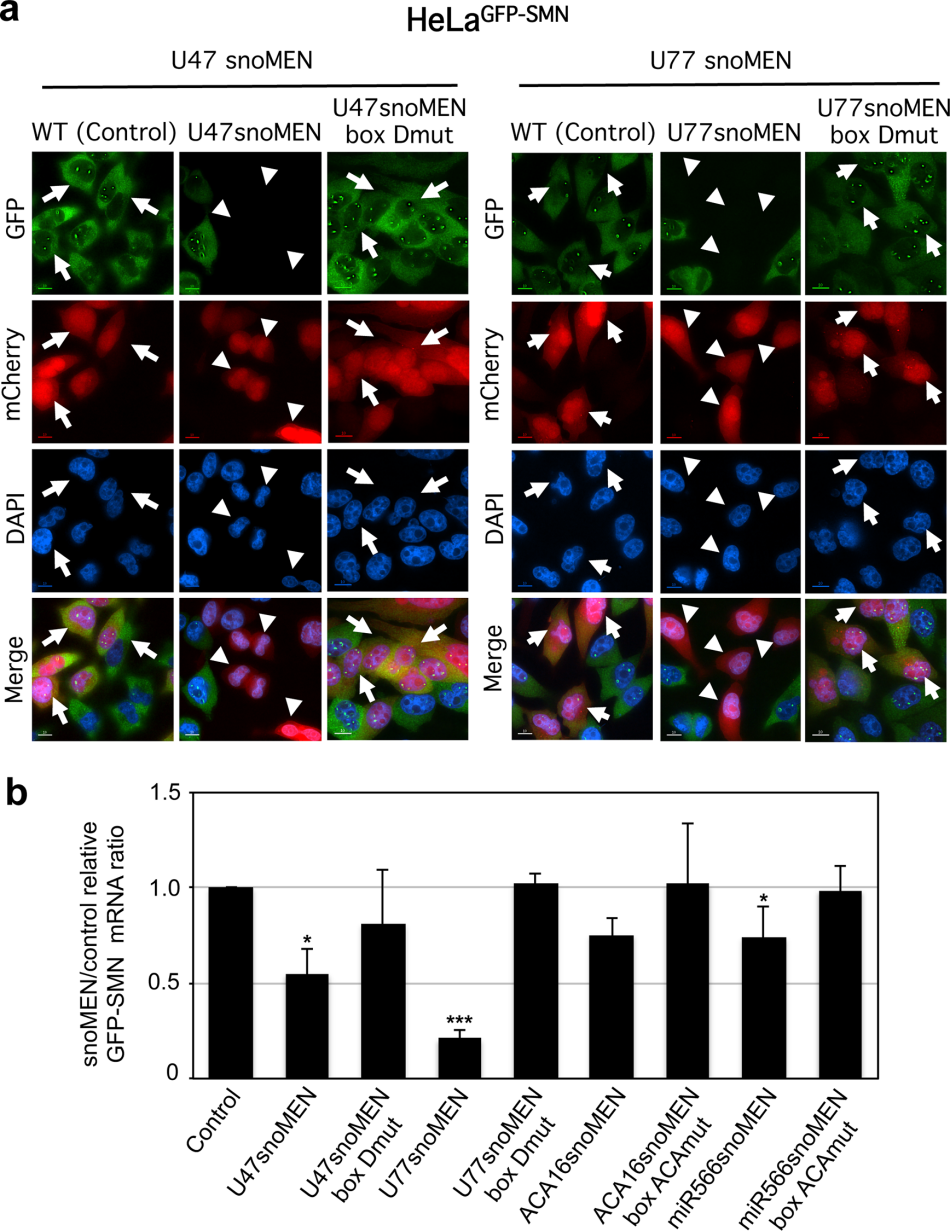
Comparison of targeted suppression of GFP fusion protein using snoMEN vectors. (**a**) The effect of U47 snoMEN (left panel) and U77 snoMEN (right panel) constructs on GFP-SMN1 expression in the HeLa^GFP-SMN^ stable cell line, which expresses GFP fused at the amino terminus of SMN1. Images show the effect of transfecting either wild type snoRNAs encoded in mCherry expression plasmid mCherry-N1 (WT, Control), expression plasmid U47/U77 snoMEN (U47snoMEN at left panel, U77snoMEN at right panel), or expression plasmid of U47/U77 boxD mutant snoMEN (U47snoMEN box Dmut at left panel, U77snoMEN box Dmut at right panel) in the HeLa^GFP-SMN^ stable cell lines. Upper panels show GFP and mCherry fluorescence signals of images recorded from fixed cells (GFP and mCherry). Lower panels show merged images combining the GFP (green) and mCherry (red) signals. Scale bar is 10 μm. The arrows indicate transfected cells and arrowheads indicate cells showing reduction of the GFP signal. (**b**) Detection of RNA levels for GFP-SMN1 by quantitative RT-PCR (qRT-PCR) following transfection of HeLa^GFP-SMN^ stable cell lines, using either the wild type snoRNAs with mCherry expression plasmid (control), expression plasmid U47 snoMEN (U47snoMEN), U47snoMEN box Dmut, U77 snoMEN (U77snoMEN), U77 snoMEN box Dmut, ACA16snoMEN (ACA16snoMEN), ACA16snoMEN box ACAmut, miR-566 snoMEN (miR566snoMEN) or miR566snoMEN box ACAmut. Equal amounts of total RNA from HeLa^GFP-SMN^ cells, extracted following snoMEN transfection, were used for qRT-PCR reactions. Graph shows the GFP-SMN1 expression ratio between control and snoMEN/mutant snoMEN transfection measured from four independent experiments. *P*-values are significant according to the Student's *t*-test; *P<0.05, **P<0.01, ***P<0.001. GFP specific primers and U3 snoRNA specific primers, as a loading control, were used for amplification.

The ability of each new snoMEN vector to modulate expression of stably expressed GFP was evaluated in HeLa cells. In each case the M box sequence of snoRNAs U47, U77, ACA16 and hsa-miR-566 precursor, had been replaced with sequences complementary to the coding sequence of GFP mRNA (**Fig 1B** and **Figs C-E** in **S1 File**). Plasmid vectors encoding mCherry linked either to wild type snoRNAs, or linked to snoMEN targeted to GFP mRNA, were transiently transfected in HeLa cell lines that stably express GFP-SMN1 (HeLa^GFP-SMN^) [17] (**Fig 4A** and **Fig F** in **S1 File**). Expression of mCherry was used as a marker for detecting transfected cells. A reduced level of GFP-SMN1 fluorescence was observed specifically in cells co-expressing mCherry and snoMEN (arrowheads), but not upon transient co-expression of controls corresponding to mCherry and wild type snoRNAs (arrow). Furthermore, a reduction in GFP-SMN1 fluorescence was not observed upon transfection with mutant snoMEN expression plasmids where the sequence of either the box D, or box ACA core motifs, was mutated (arrows).

**Fig 5:**
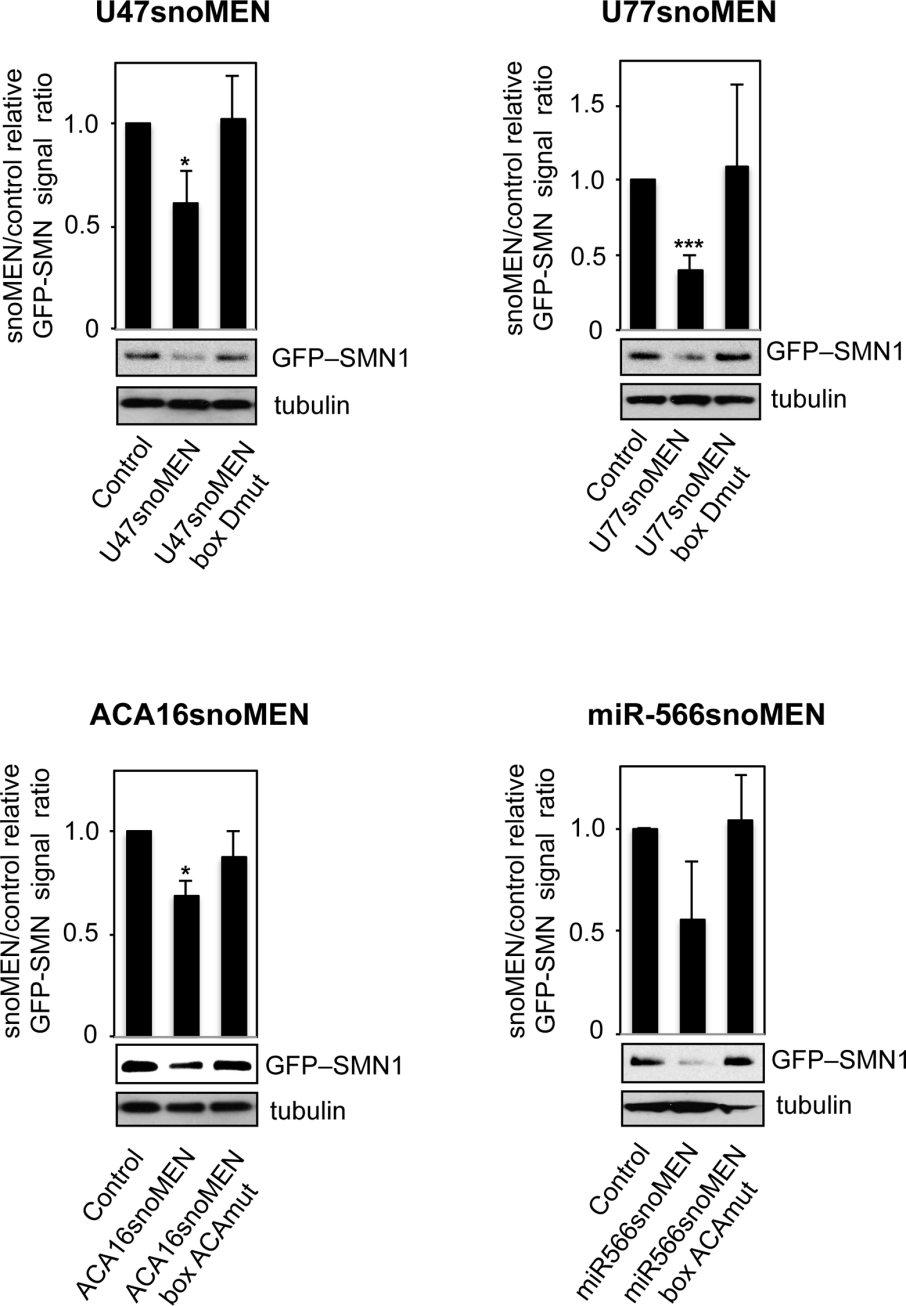
Western blot analysis for snoMEN knockdown. Knock-down efficiency of each of the snoMEN plasmids targeted to GFP (U47snoMEN, U77snoMEN, ACA16snoMEN and miR-566snoMEN at the top of each panel) was tested by western blotting. Detection of protein levels for GFP-SMN1 following transfection of HeLa^GFP-SMN^ stable cell lines, using either mCherry and wild type snoRNA expression plasmid (control), snoMEN expression plasmid (U47snoMEN, U77snoMEN, ACA16snoMEN and miR566snoMEN) and mutant snoMEN expression plasmid (U47snoMEN box Dmut, U77snoMEN box Dmut, ACA16snoMEN box Dmut and miR566snoMEN box Dmut). An equivalent amount of HeLa^GFP-SMN^ extract was loaded for each lane and the proteins separated by SDS PAGE, electroblotted onto membrane and probed both with a monoclonal anti-GFP antibody and with anti-tubulin as a loading control. Graph shows average ratio of GFP-SMN1 signal intensity, normalised using the tubulin signal, which calculated from three independent experiments. *P*-values are significant according to the Student's *t*-test; *P<0.05, **P<0.01, ***P<0.001.

Analysis of GFP-SMN1 mRNA levels, as determined using quantitative RT-PCR assays, yielded similar results (**Fig 4B**). The newly established snoMEN reduced GFP-SMN1 expression in the HeLa^GFP-SMN^ stable cell line compared with the GFP-SMN1 mRNA expression levels of the control snoRNA, with varying efficiency (U47snoMEN & U77snoMEN: >50%, ACA16snoMEN & miR-566snoMEN: ~20%) (**Fig 4B** Control). Control experiments showed that neither GFP-SMN1 mRNA levels (**Fig 4B**), nor protein levels (**Fig 5**), were reduced by transfecting the same cells with mutant snoMEN expression plasmids. Compared with previous data for HBII-180C snoMEN targeted to GFP-SMN1 ([9] also see below), these results indicate that two of the novel box C/D snoRNAs, i.e. U47 and U77, outperform the knockdown level of the previous HBII-180C snoMEN RNA. As the U47 snoRNA is shorter than U77 and it is easier to manipulate its sequence, we focussed on characterising U47 in detail to evaluate its use as a novel snoMEN vector for targeted gene suppression.

### Multiplex U47snoMEN vector transcripts

**Fig 6:**
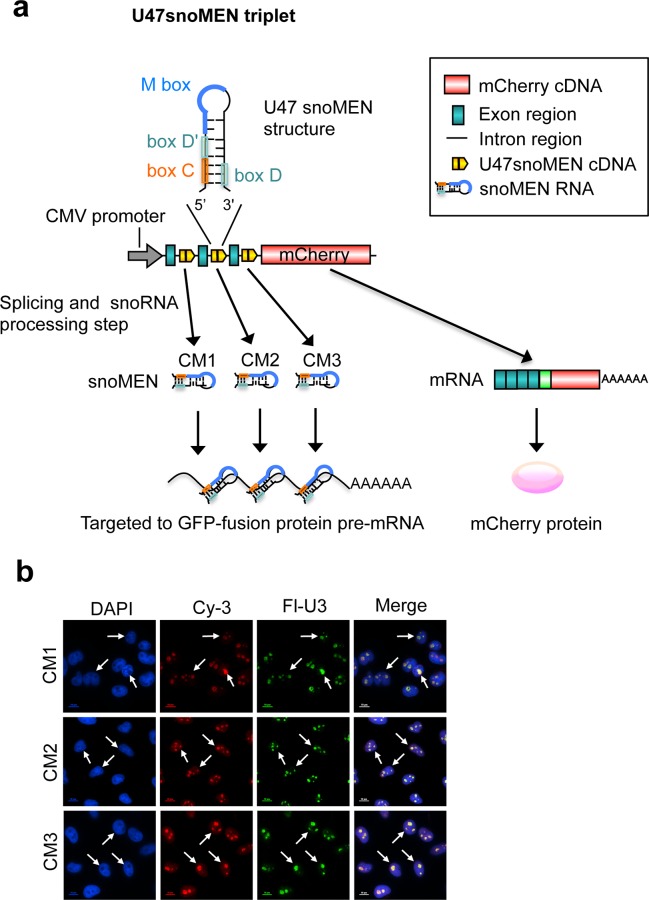
Triple U47 snoMEN vector. (**a**) A triple U47 snoMEN construct, which encodes three U47 snoMEN targeting different GFP regions (CM1-3), was constructed. (**b**) Fluorescence *In Situ* Hybridisation using Cy3 labelled U47 snoMEN specific probes (Cy3) showed nuclear localization and nucleolar accumulation. A U3 snoRNA specific probe was also co-hybridised as a nucleolar marker (Fl, Green). DNA is stained by DAPI. Scale bar is 10 μm. Arrow shows the nucleolus.

**Fig 7:**
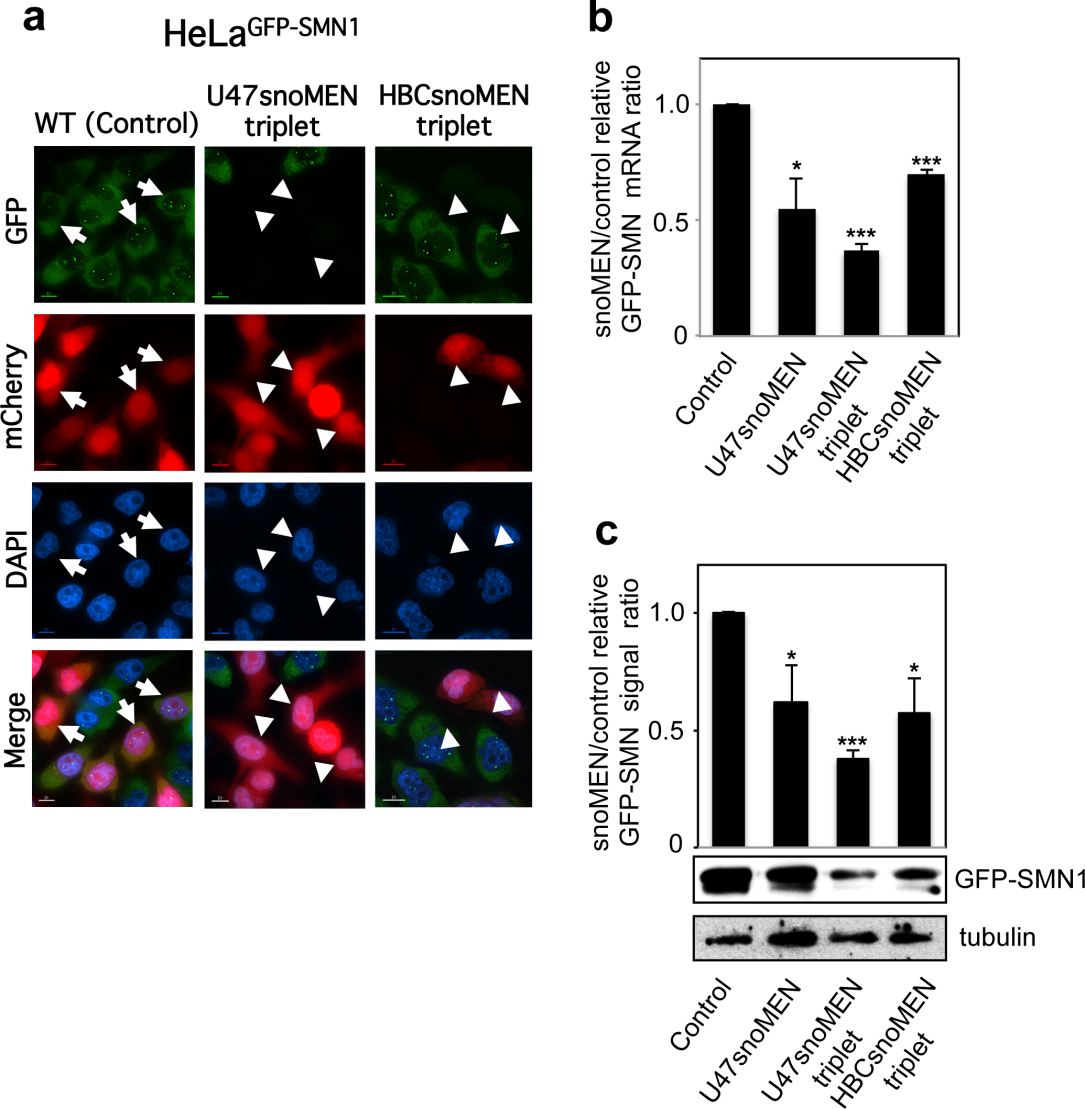
Gene knock-down using snoMEN triplet vector. (**a**) This shows the same experiment as in **Fig 4A**, except that the triple U47 snoMEN (U47snoMEN triplet) and triple HBII-180C snoMEN (HBCsnoMEN triplet) were transfected. Scale bar is 10 μm. (**b**) This is the same quantification analysis as shown in **Fig 4B**, except that the triple U47 snoMEN (U47snoMEN triplet) and triple HBII-180C snoMEN (HBCsnoMEN triplet) were transfected. Graph shows the GFP-SMN1 expression ratio between control and triple snoMEN transfections, measured from four independent experiments. *P*-values are significant according to the Student's *t*-test; *P<0.05, **P<0.01, ***P<0.001. GFP specific primers and U3 snoRNA specific primers, as a loading control, were used for amplification. (**c**) This is the same quantification analysis as shown in **Fig 5**, except that the triple U47 snoMEN (U47snoMEN triplet) and triple HBII-180C snoMEN (HBCsnoMEN triplet) were transfected. Graph shows average ratio of GFP-SMN1 signal intensity, normalised using the tubulin signal, which was calculated from three independent experiments. *P*-values are significant according to the Student's *t*-test; *P<0.05, **P<0.01, ***P<0.001.

We next investigated the design of vectors that can deliver multiple U47 snoMEN RNAs from a single transcript. Thus, a triplet U47snoMEN vector was constructed that encodes mCherry as a transfection marker and three M box-modified U47 snoRNAs, each targeted to different positions in the GFP mRNA sequence (**Fig 6A**). FISH analysis, using specific probes for each U47snoMEN (**Fig 6B** CM1-3), showed that all three M box-modified U47 snoRNAs were expressed following transfection and localised in the nucleus in a similar pattern to wild type U47 snoRNA (**Fig 2**). As shown in **Fig 7**, transient transfection of the triple U47snoMEN vector (U47snoMEN triplet) showed knock-down of fluorescence in cells expressing GFP-fusion proteins, as did the HBCsnoMEN triplet positive control, which corresponds to the original HBII-180C snoMEN targeted the same GFP sequences as the U47snoMEN triplet (**Fig 7A** arrowhead). Quantification of both relative mRNA and protein levels tested by qRT-PCR (**Fig 7B**) and western blot (**Fig 7C**), respectively, showed that the knock-down efficiency was increased using the triple U47snoMEN vector, as compared either with vectors expressing a single U47 snoMEN, or with the vectors expressing triple HBII-180C snoMEN (**Fig 7B** and **7C**).

### Enhanced protein replacement using the U47snoMEN vector

**Fig 8:**
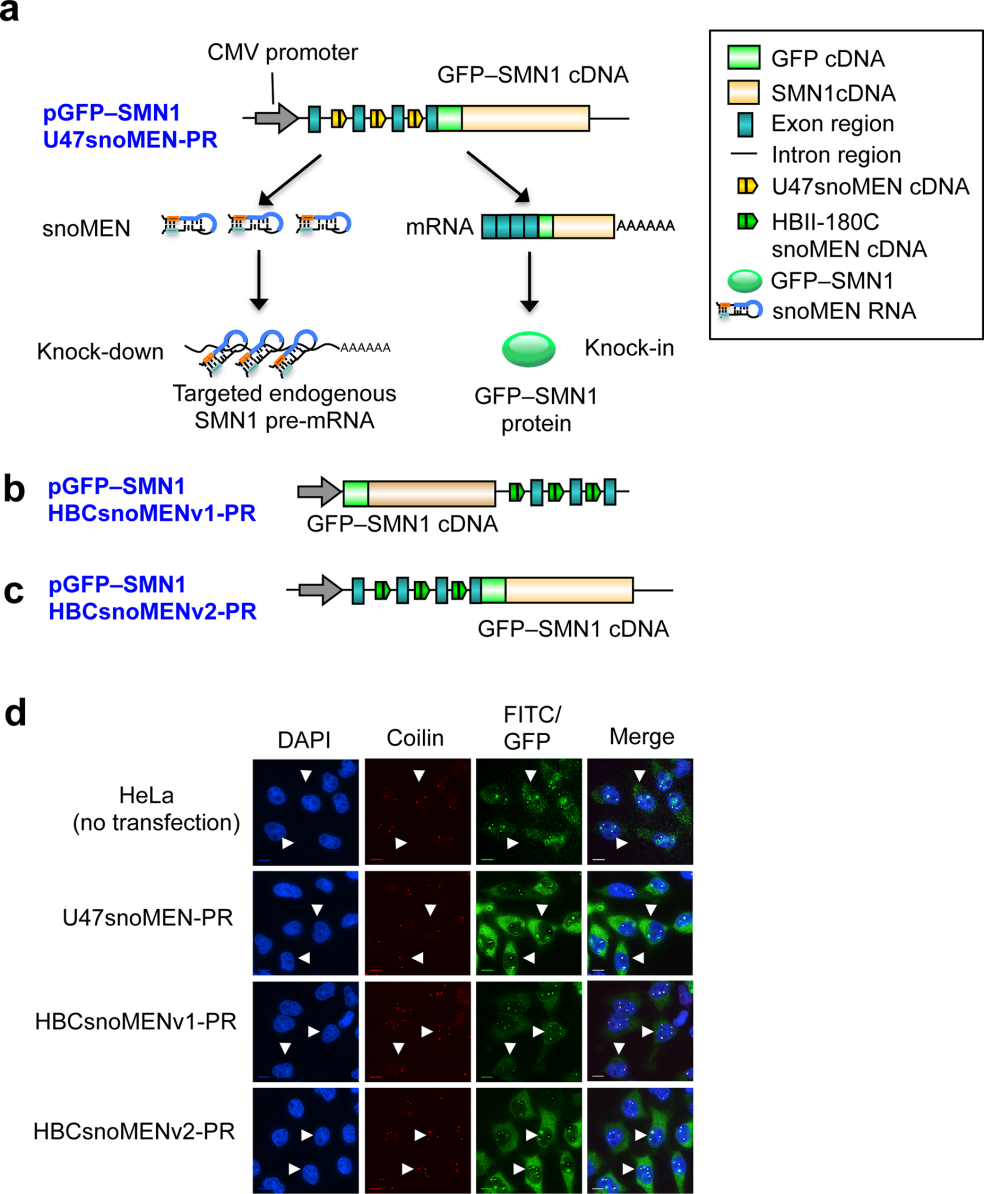
Protein replacement of endogenous SMN1 protein using advanced snoMEN. (**a**)-(**c**) Structures are shown for targeted endogenous SMN1 protein replacement plasmid, using either U47snoMEN (a: pGFP-SMN1 U47snoMEN-PR), or SMN1 protein replacement plasmid using HBII-180C snoMEN (b: pGFP-SMN1 HBCsnoMENv1-PR, c: pGFP-SMN1 HBCsnoMENv2-PR). These constructs have three snoMEN sequences, as in the triple U47 snoMEN plasmid (**Fig 6A**), except that here the M box sequences are complementary to sequences in the endogenous SMN1 pre-mRNA. (**d**) Localisation analysis of endogenous SMN1 (HeLa: no transfection) and GFP-SMN1 protein (arrowheads), in HeLa cells transiently transfected with the U47snoMEN-PR, HBCsnoMENv1-PR and HBCsnoMENv2-PR plasmids, respectively. Images show the localisation patterns of DNA (DAPI, Blue), endogenous SMN1 (FITC)/GFP-SMN1 proteins (GFP, Green) and endogenous p80 coilin protein (Red), stained as a Cajal body marker. Scale bar is 10 μm.

Next, we tested the knock-down efficiency when targeting U47 snoMEN to an endogenous gene, rather than to GFP. For this we designed a protein replacement vector, pGFP-SMN1 U47snoMEN-PR (**Fig 8A**), expressing M box-modified U47 snoRNAs targeted to endogenous SMN1 pre-mRNA specific sequences, based on the U47snoMEN triplet plasmid (**Fig 6A**). Plasmid pGFP-SMN1HBCsnoMENv1-PR [7] (**Fig 8B**), encodes a GFP-SMN1 fusion protein and three M box modified HBII-180C snoRNAs targeted to the same sequence region as pGFP-SMN1 U47snoMEN-PR, allowing a direct comparison of relative knock-down efficiencies. To optimise the vector design, we also constructed the plasmid pGFP-SMN1HBCsnoMENv2-PR (**Fig 8C**). This encodes the same HBII-180C snoMEN triplet as pGFP-SMN1HBCsnoMENv1-PR, but here with the snoMEN sequences upstream (i.e., 5’) of the GFP-SMN1 fusion protein, to test whether this arrangement affected the efficiency of protein expression.

**Fig 9:**
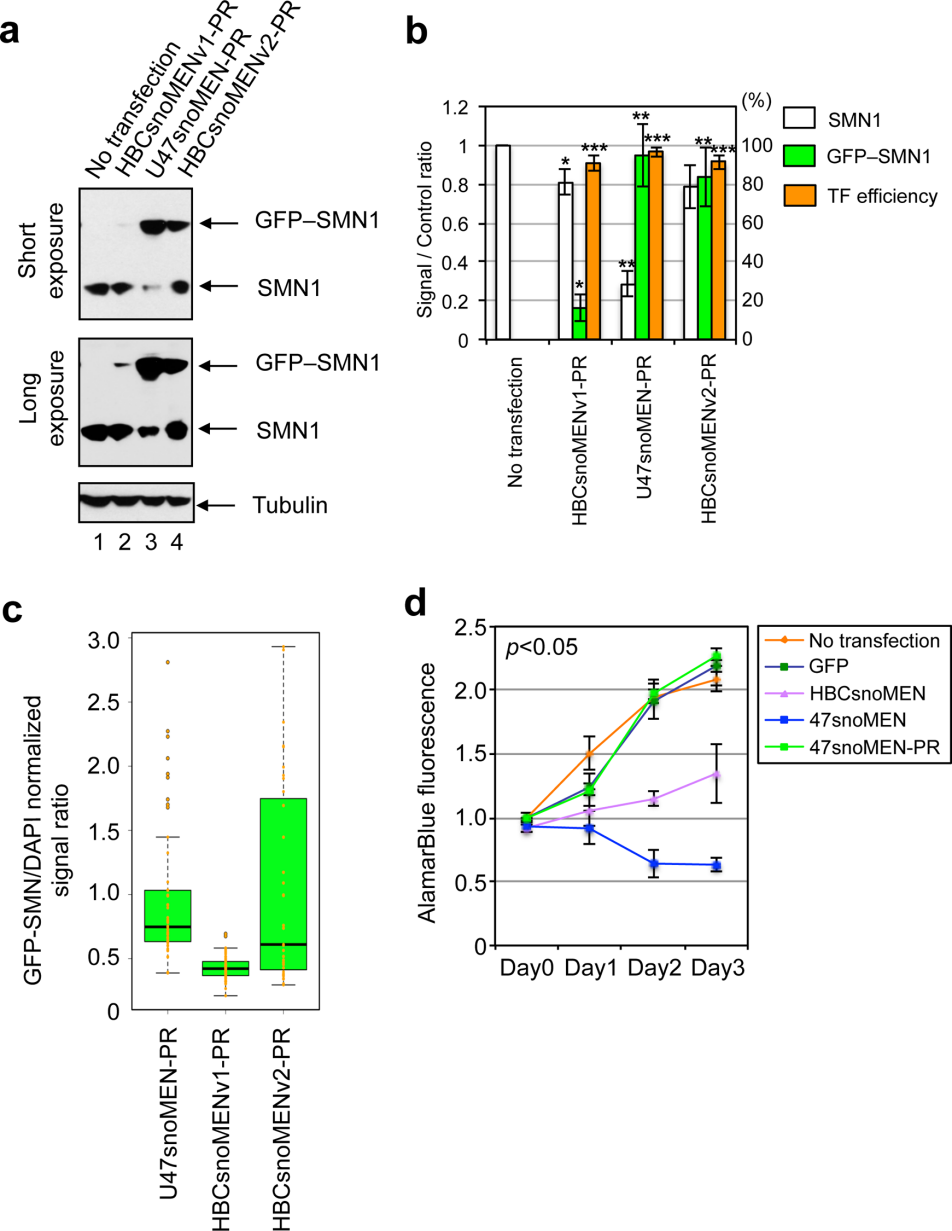
Comparison of protein-replacement efficiency. (**a**) Expression level of endogenous SMN1 and GFP-SMN1 were measured by western blot analysis. Images show an example of a western blot, with shorter exposure in the upper panel and longer exposure in the lower panel. An equivalent amount of total cell extract from HeLa cells was loaded for each lane and the proteins separated by SDS PAGE, electroblotted and probed both with a monoclonal anti-SMN1 antibody and with an anti-tubulin antibody as a loading control. (**b**) The graph shows average signal intensity and standard deviation for three independent experiments from HeLa cells transiently transfected with U47snoMEN-PR, HBCsnoMENv1-PR and HBCsnoMENv2-PR plasmids, as shown in **Fig 8D**. The SMN1/GFP-SMN1 signal ratio was normalised to the tubulin signal. Ratios were calculated by comparison with endogenous SMN1 signals in control (No transfection), untransfected cells. Transfection efficiency was determined by counting the number of GFP positive cells in 100 randomly selected cells (TF efficiency: orange bar). *P*-values are significant according to the Student's *t*-test; *P<0.05, **P<0.01, ***P<0.001. (**c**) Graph shows a box plot of GFP-SMN1/DAPI signal ratio comparing transfected U47snoMEN-PR, HBCsnoMENv1-PR and HBCsnoMENv2-PR plasmids. Signals were calculated from 50 randomly selected cells. (**d**) Results of proliferation/cytotoxicity assays comparing control and SMN1 protein replacement cells. A GFP alone expression vector was transiently transfected into HeLa cells as a negative control. The effects of transient transfection with vectors expressing either endogenous SMN1 targeted 47snoMEN, HBCsnoMEN without SMN1 expression (47snoMEN and HBCsnoMEN), or pGFPSMN1-U47snoMEN-PR (47snoMEN-PR), were also measured. Note, both 47snoMEN and HBCsnoMEN showed cytotoxic effects when transiently transfected into HeLa cells, however, this cytotoxicity was rescued by transfection with the 47snoMEN-PR vector. *P*-values are significant according to the Student's *t*-test (*P*<0.05).

Transient transfection of U47snoMEN-PR, HBCsnoMENv1-PR and HBCsnoMENv2-PR all showed expression of GFP-SMN1, with similar localisation patterns, closely resembling the localisation of endogenous SMN1, as confirmed by co-localisation with the Cajal body marker coilin [17] (**Fig 8D** arrowhead). Western blot analysis was performed to measure the ratio of GFP-SMN1/endogenous SMN1 protein replacement for each of the snoMEN vectors (**Fig 9A**). Both HBII-180C snoMEN expression plasmids, i.e. HBCsnoMENv1-PR and HBCsnoMENv2-PR, show a reduction in the levels of endogenous SMN1 and either ~20% (HBCsnoMENv1-PR), or ~80% (HBCsnoMENv2-PR) knock-in of GFP-SMN1 proteins (**Fig 9A** and **9B**, lanes 2 and 4, graphs HBCsnoMENv1-PR and HBCsnoMENv2-PR white and green bars). This showed that expression of the ‘knock-in’ protein is increased when it is located in the transcript downstream (i.e. 3’) of the three snoMEN RNAs. The U47snoMEN-PR expression plasmid, meanwhile, showed more efficient (~70%) reduction of the endogenous SMN1 protein and a correspondingly higher knock-in level of GFP-SMN1 (**Fig 9A** and **9B**, lane 3, graphs U47snoMEN-PR white and green bars). Analysis of transfection efficiency indicated that ~95% of HeLa cells were transfected with each of the respective snoMEN-PR plasmids (**Fig 9B** orange bars in graph).

The level of GFP-SMN1 fluorescence was compared between the HeLa cells transfected with either U47snoMEN-PR, HBCsnoMENv1-PR, or HBCsnoMENv2-PR plasmids, respectively (**Fig 9C**). This shows higher levels of GFP-SMN1 expression in cells transfected with either U47snoMEN-PR, or HBCsnoMENv2-PR plasmids, as compared with the HBCsnoMENv1-PR plasmid (**Fig 9C**). Overall, these results show that the levels of endogenous SMN1 protein can be suppressed more than three times more efficiently using the M box-modified U47 snoRNAs, (i.e. 47snoMEN), as compared with the previously characterised HBII-180C snoMEN. The data also show that ~5-fold increase in protein expression efficiency can be achieved by locating all the snoMEN RNAs upstream of the coding sequence of the ‘knock-in’ protein in the vector transcript.

To test the phenotypic effect of SMN1 knock-down by U47snoMEN, the growth rate of cells was examined by a proliferation assay (**Fig 9D**). SMN1 depletion is known to cause apoptosis [17]. Transient transfection of either GFP–C1 (GFP: expressing GFP protein alone), or pGFP–SMN1U47snoMEN-PR (47snoMEN-PR), did not cause either cytotoxicity, or growth suppression. However, transient transfection of the SMN1-targeted U47snoMEN expression plasmid (47snoMEN), resulted in pronounced cytotoxicity that was more efficient than the degree of cytotoxicity caused by transient transfection with the former version of the snoMEN vector (HBCsnoMEN) (**Fig 9D**). These results show that the cytotoxicity caused by SMN1 depletion was rescued by snoMEN protein replacement and further confirm the increased efficiency provided by the new U47snoMEN vector.

### Establishment of stable protein replacement cell lines using 47snoMEN-PR vectors

**Fig 10:**
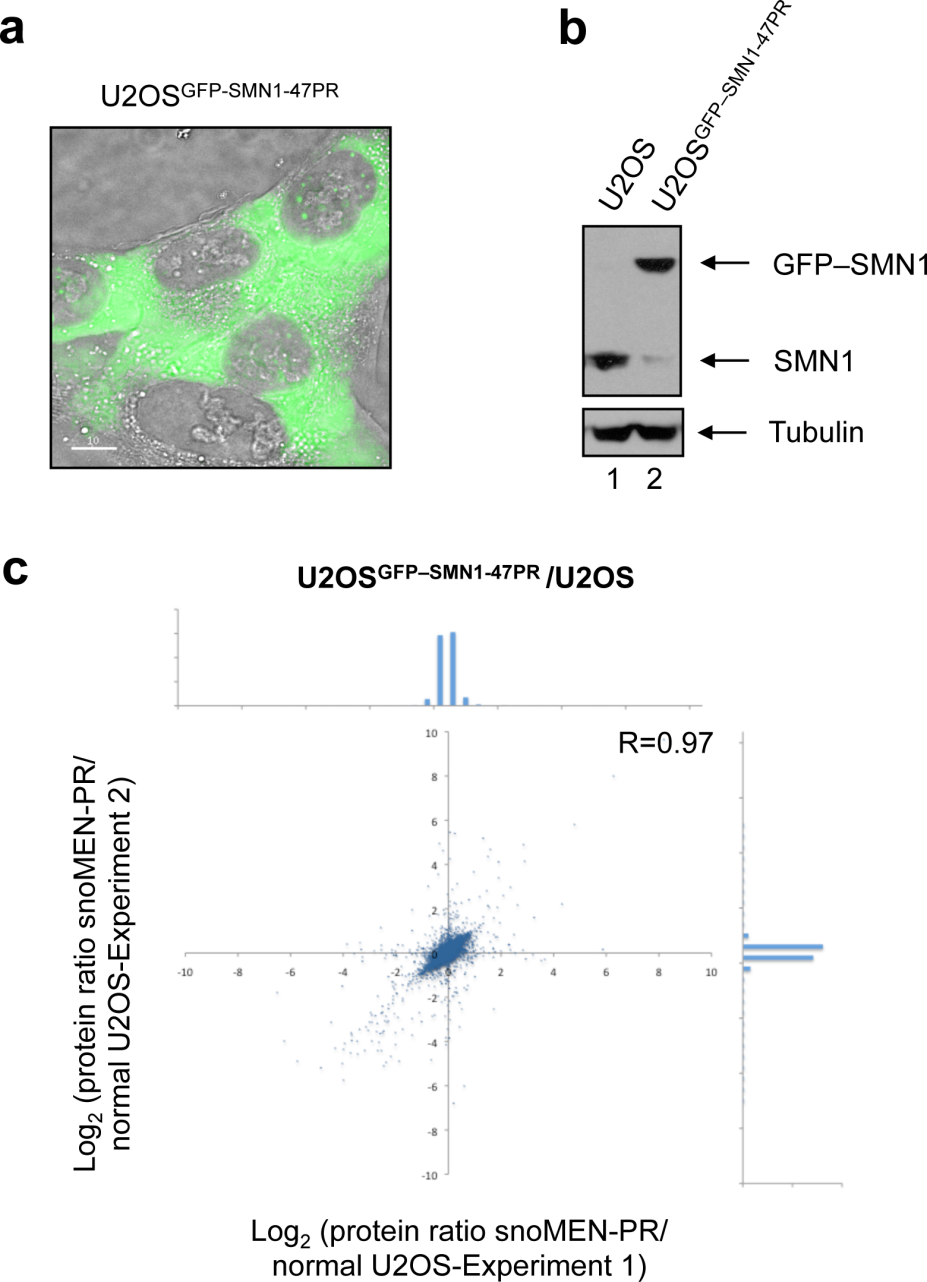
Establishment of human protein replacement stable cell lines using 47snoMEN. (**a**) Images of protein replacement stable cell lines. Expression of FP proteins was confirmed by fluorescence imaging. Bar is 10 μm. (**b**) Expression levels of endogenous SMN1 and GFP-SMN1 proteins were measured by western blot analysis. An equivalent amount of total cell extract from U2OS and U2OS^GFP–SMN1-47PR^ stable cells was loaded for each lane and the proteins separated by SDS PAGE, electroblotted and probed both with a monoclonal anti-SMN1 antibody and with an anti-tubulin antibody as a loading control. (**c**) Gene-expression profiles were compared between U2OS and U2OS^GFP–SMN1-47PR^ cells by quantitative mass spectrometry analysis. Comparison of protein expression levels detected by mass spectrometry for U2OS and U2OS^GFP–SMN1-47PR^ cells. Each SILAC experiment was independently repeated three times. Correlation between protein ratios of SILAC experiments visualised on a 2D logarithmic graph for all detected proteins, identified as previously described [44, 45]. On the x and y axis, log_2_ (H/L ratio) correlates with the enrichment in U2OS cells and U2OS^GFP–SMN1-47PR^ cells for experiment 1 and experiment 2, respectively. Graph shows a distribution pattern of plot numbers. SILAC ratio values of labelled proteins are listed in **S2 Data**.

We next established a stable human protein replacement U2OS cell line for expression of the GFP-SMN1 protein, i.e., U2OS^GFP–SMN1-47PR^ using the pGFP-SMN1 U47snoMEN-PR vector (**Fig 10A**). Consistent with the improvement in vector design shown by the previous transient transfection assays, this new stable cell line created using 47snoMEN showed greatly reduced expression of the endogenous SMN1 protein and a more efficient replacement with GFP-SMN1, as compared with a similar GFP-SMN1 protein replacement stable cell line established using the previous snoMEN protein replacement vector (i.e., U2OS^GFP–SMN1-PR^) [9] (**Fig 10B**). Proteomic analysis by SILAC was carried out to analyse the effect of constitutive expression of the pGFP-SMN1 U47snoMEN-PR vector by comparing the steady state proteomes of the new U2OS^GFP–SMN1-47PR^ stable cell line with the parental U2OS cell line (**Fig 10C**). This showed that ~96% of the >7,200 proteins detected by MS were expressed at similar levels (<2 fold difference) in both cell lines.

**Fig 11:**
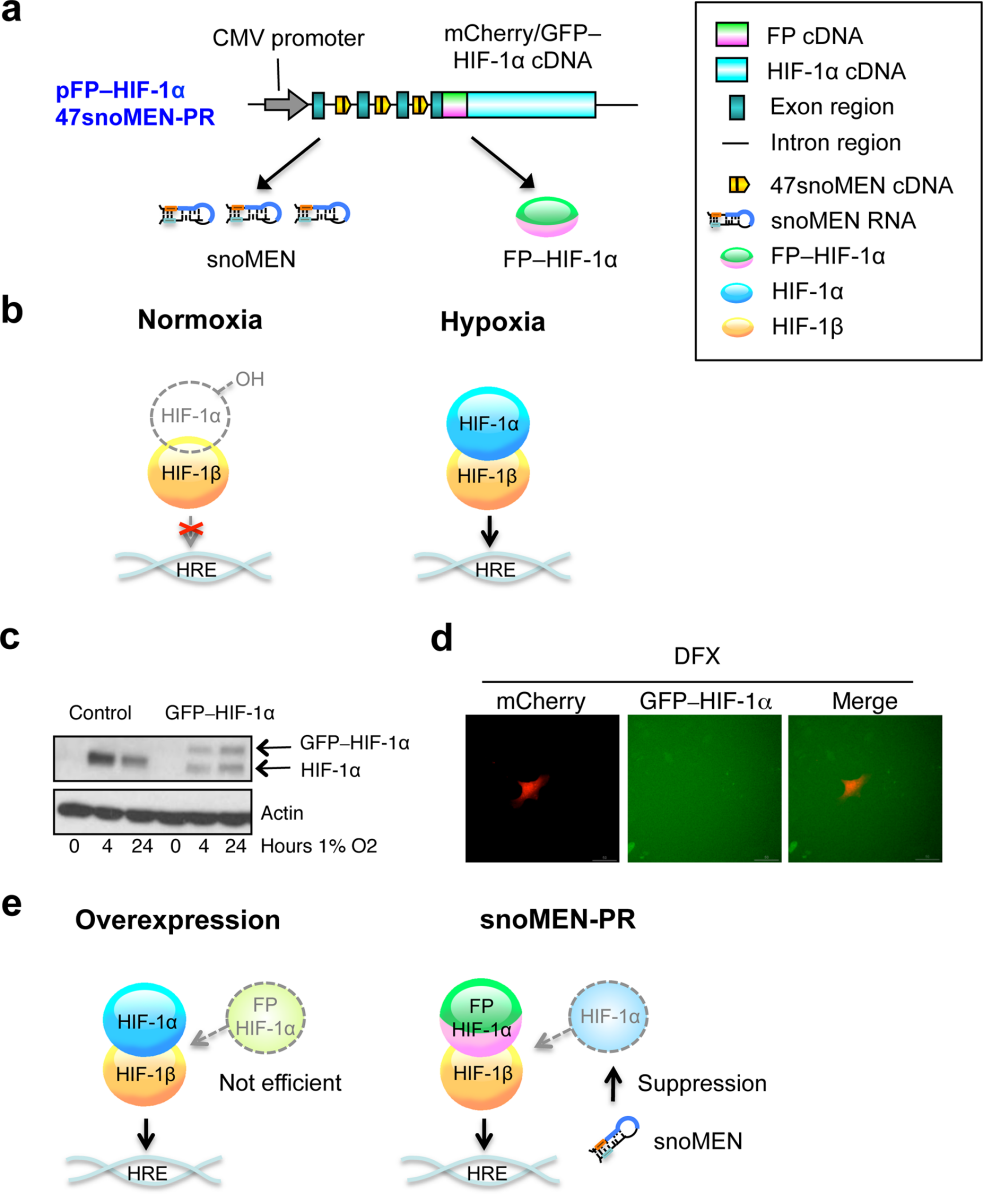
Protein replacement of endogenous HIF-1α protein using 47snoMEN. (**a**) Structures for the targeted endogenous HIF-1α protein replacement plasmid using U47snoMEN. (**b**) Schematic diagram of endogenous HIF complex. (**c**) U2OS and U2OS GFP–HIF-1α stable cell lines were exposed to hypoxia prior to lysis and analysed by Western blot for HIF-1α levels. (**d**) U2OS GFP–HIF-1α stable cell lines were transfected with mCherry and treated with DFX for 24 hours. Cells were analysed by imaging for the levels of HIF-1α (**e**) Schematic diagram of external HIF-1α protein.

Given the improved performance of the new U47snoMEN vectors, we next sought to establish a stable knock-in cell line for GFP–HIF-1α (hypoxia-inducible factor-1 alpha), a protein for which it has previously proven difficult to establish stable cell lines (**Fig 11A**). HIF-1α is a transcription factor that is induced under low oxygen conditions, termed Hypoxia. HIF-1α is hydroxylated by PHD (prolyl hydroxylase) enzymes under normal oxygen conditions (Normoxia) and is degraded by hydroxylation-dependent proteasomal degradation (**Fig 11B**) [18]. In hypoxia, PHDs show reduced activity, leading to stabilisation of HIF-1α, which can then form a heterodimer with HIF-1β, which is constitutively expressed under both Normoxia and Hypoxia conditions.

Previous attempts at creating GFP–HIF-1α stable cell lines have been unsuccessful, as cells are unable to retain high levels of this protein and still proliferate. Indeed, HeLa cells stably transfected with GFP–HIF-1α showed reduced expression of endogenous HIF-1α to accommodate similar levels of the exogenous tagged version (**Fig 11C**). These cells were thus unsuitable for imaging due to the low GFP signal (**Fig 11D**). The lack of a convenient system for efficient protein replacement has thus presented an obstacle to establishing a FP tagged HIF-1α stable cell line (**Fig 11E**).

**Fig 12:**
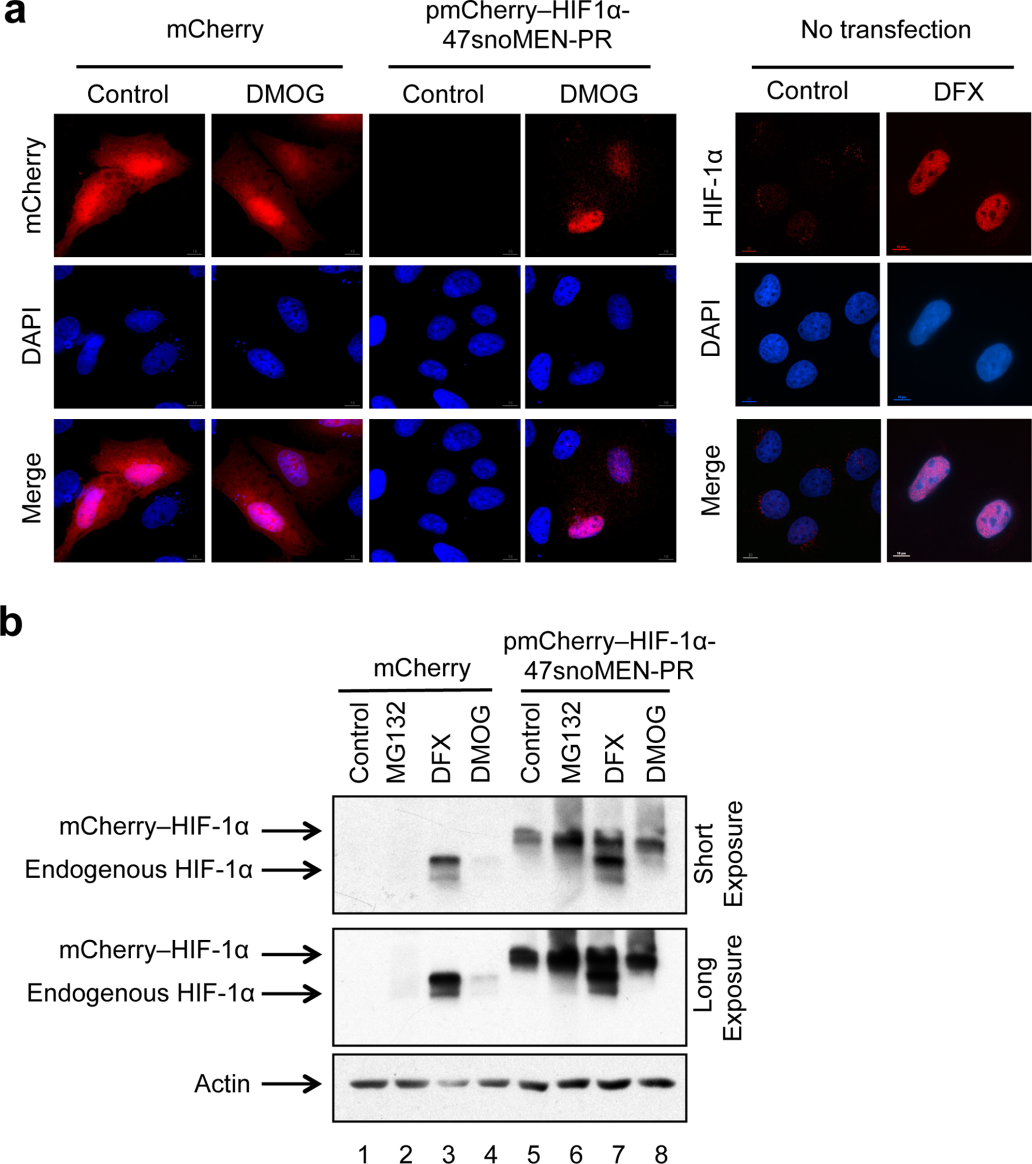
Protein replacement of endogenous HIF-1α protein by transient transfection. (**a**) Localisation analysis of endogenous HIF-1α (no transfection) and mCherry–HIF-1α protein (pmCherry–HIF1α-U47snoMEN-PR) in U2OS cells transiently transfected with the U47snoMEN-PR plasmid. HIF-1α was stabilised by DMOG/DFX treatments. While DFX is an iron chelator, DMOG, is a 2-oxoglurate analogue that blocks enzymes that require this as co-factor, making DMOG slightly more specific. The difference in efficiency in stabilizing HIF-1α reflects the concentrations of each inhibitor used. Images show localisation patterns of DNA (DAPI, Blue) and endogenous HIF-1α / mCherry–HIF-1α proteins (Red), respectively. A plasmid vector coding for mCherry alone was also transiently transfected as a negative control. Scale bar is 10 μm. (**b**) Expression levels of endogenous HIF-1α and mCherry–HIF-1α were measured by western blot analysis. A plasmid vector coding for mCherry alone was also transiently transfected as a negative control. The actin signal was used as a loading control.

To address this using the new 47snoMEN-PR vector system, we constructed the vector pmCherry–HIF-1α 47snoMEN-PR, which encodes three 47 snoMEN that target three separate regions of HIF-1α pre-mRNA cloned upstream of mCherry–HIF-1α cDNA (**Fig 11A**). This 47snoMEN-PR vector was initially evaluated by transiently transfecting U2OS cells (**Fig 12**). Transient transfection of pmCherry–HIF-1α 47snoMEN-PR showed expression of mCherry–HIF-1α with a similar nuclear localisation pattern to that seen with endogenous HIF-1α (**Fig 12A**). The expression of mCherry–HIF-1α was also confirmed by Western blot analysis (**Fig 12B** lanes 5–8). The expression of mCherry–HIF-1α was stabilized by MG132 treatment, which inhibits proteasome activity (**Fig 12B** lanes 5 and 6) and was also increased by treatment with DFX and DMOG (**Fig 12B** lanes 5, 7 and 8), which are inhibitors of PHD enzymes and thus mimics for hypoxia. These results suggested that the 47snoMEN-PR vector could potentially allow establishment a HIF-1α protein replacement stable cell line that efficiently express FP tagged–HIF-1α.

**Fig 13:**
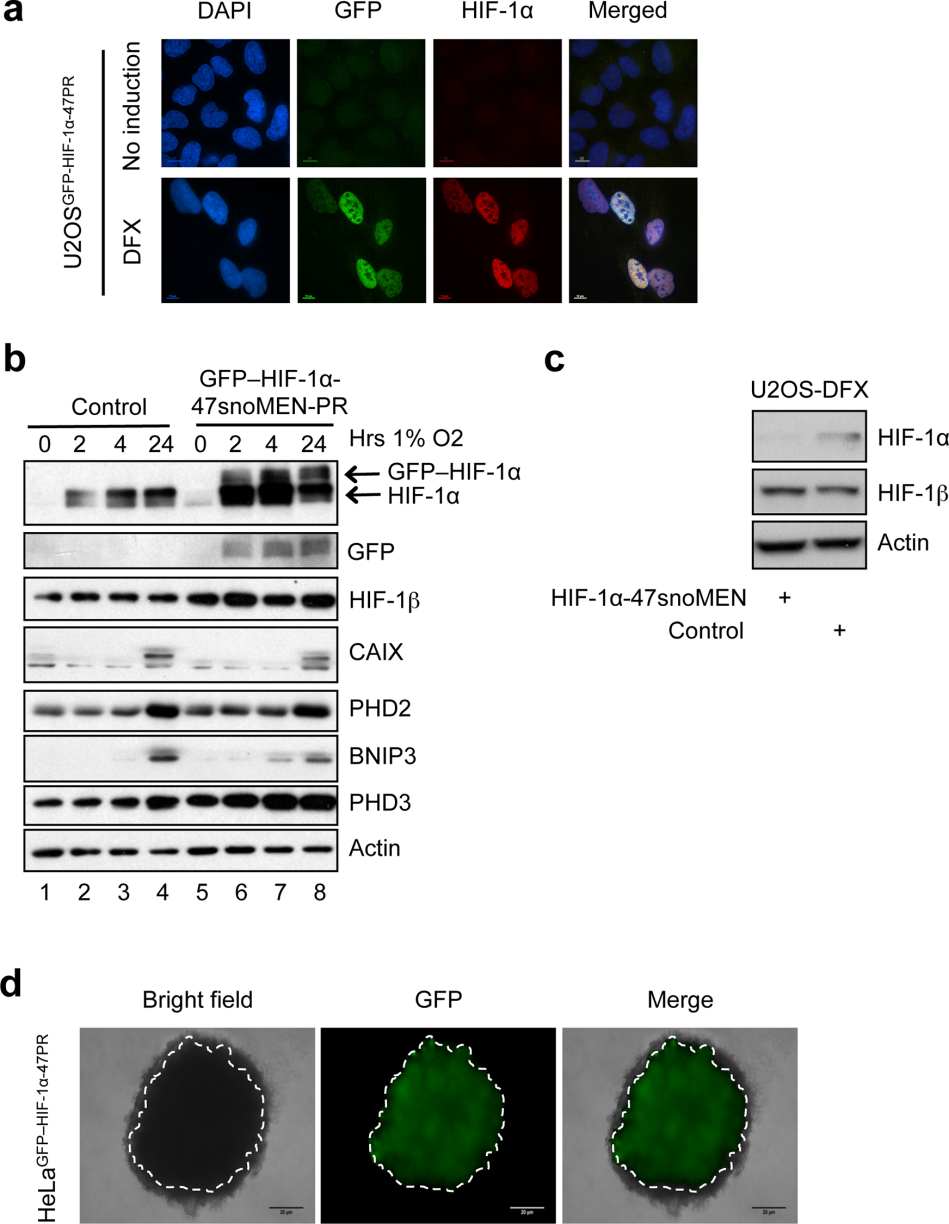
Establishment of a human protein replacement stable cell line using 47snoMEN. (**a**) Images of protein replacement stable cell lines (U2OS^GFP–HIF-1α-47PR^). Expression of FP proteins was confirmed by fluorescence imaging with DFX treatment. Bar is 10 μm. (**b**) U2OS and U2OS^GFP–HIF-1α-47PR^ cells were exposed to 1% O_2_ for the indicated periods of time prior to lysis. Cell extracts were analysed by western blot using the indicated antibodies. (**c**) U2OS cells were transfected with 1 μg of control and HIF-1α -47snoMEN vectors for 48 hours prior to lysis. DFX was added for the last 24 hours. Cell extracts were analysed by western blot using the indicated antibodies. (**d**) 3D culture analysis. HeLa^GFP–HIF-1α-47PR^ cells were cultured in lipidure coated plates to induce spheroid formation. HIF-1α is visible in the core of the spheroid revealing hypoxia. Bar is 10 μm.

Next, we established two different human protein replacement stable cell lines, U2OS^GFP–HIF-1α-47PR^ and HeLa^GFP–HIF-1α-47PR^, using the pGFP–HIF-1α 47snoMEN-PR plasmid vector, which encodes three 47snoMEN that target three separate regions of endogenous HIF-1α pre-mRNA and a cDNA encoding GFP–HIF-1α that does not include the sequences targeted by the snoMEN (**Fig 11A**). Both of these stable cell lines created using 47snoMEN vectors showed clear expression of GFP–HIF-1α in the presence of the hypoxia mimicking agent DFX (**Fig 13A** and **13D**). We therefore tested how these cells responded to real hypoxia, by exposing parental U2OS and U2OS^GFP–HIF-1α-47PR^ cells to 1% oxygen for different times. Analysis of the resulting cell extracts revealed that, upon hypoxia, HIF-1α was stabilised and HIF-dependent target genes were induced to similar levels as seen in the parental U2OS cells (**Fig 13B**). We also tested the level of knock-down achieved by the HIF-1α-snoMEN vector, which induces a clear, albeit not complete, reduction in the levels HIF-1α (**Fig 13C**). In addition, we tested if these cells were suitable to detect physiological hypoxia, as encountered in 3-D cell culture models. To this end 3-D cultures of HeLa^GFP–HIF-1α-47PR^ cells were grown and levels of GFP–HIF-1α were analysed by fluorescence imaging (**Fig 13D**). Importantly, we were able to detect GFP–HIF-1α in the core of the 3-D culture of HeLa^GFP–HIF-1α**-**47PR^ cells, which correlated with the hypoxic core in the centre of tissue when cells are grown in this type of 3-D culture.

These results demonstrate that the pGFP–HIF-1α 47snoMEN-PR cell lines provide the first reagents suitable for studying processes leading to HIF-1α stabilisation, particularly under live cell imaging conditions where previously only transient overexpression experiments have been possible.

## Discussion

In this study we have characterised human snoRNAs that co-purify with nucleoli isolated from HeLa cells and evaluated their use in mammalian cells for targeted gene knock-down in the snoMEN vector system. Previously, we have shown that the Box C/D nucleolar snoRNA, HBII-180C, can be manipulated to create an internal region (M box), that, when complementary to either mRNA or pre-mRNA sequences, can result in reduced expression (i.e. knock-down), of both the targeted mRNA and cognate protein [7]. We have shown that snoMEN RNAs, based on engineered versions of the HBII-180C snoRNA backbone, can be expressed from either plasmid, or lentiviral vectors and can be used to knock-down targeted microRNAs, as well as protein coding transcripts [8]. Furthermore, we have shown that snoMEN vectors can be used to establish stable human cell lines that simultaneously ‘knock-down’ the levels of a targeted, endogenous protein and ‘knock-in’ expression of either a tagged, or mutant form of the same protein, using a single vector transcript [9]. Here we have aimed to improve the overall efficiency of the snoMEN vector system by characterising alternative snoRNA backbones suitable for use in targeted gene knock-down and protein replacement studies. Analysis of both human box C/D and box H/ACA snoRNAs led us to identify the U47 box C/D snoRNA as an effective backbone for creating snoMEN vectors. We show that the M box region of U47 snoMEN can be modified to target knock-down of either FP-tagged fusion proteins, or endogenous cell proteins. We characterise new snoMEN vectors based on multiplexed U47 snoMEN that provide more efficient knock-down and protein replacement than the previous vectors based on the use of snoRNA HBII-180C. Thanks to the improved efficiency of the new vectors, we have now been able to establish stable human cell lines that knock-in GFP-tagged HIF-1α, which had not been possible using alternative technologies.

Previous studies on nucleolar snoRNAs have extensively characterised their essential role in catalysing the site specific, post-transcriptional modification of rRNA and other non-coding RNAs, including tRNAs and snRNAs. In the case of box C/D snoRNAs, which catalyse 2’-O-ribose methylation by the conserved snoRNP protein fibrillarin, they have a guide region located immediately 5’ to either the box D, or D’ regions, that is complementary to the RNA target site for methylation [4, 5] (For example see **Fig 1A** Green lines). Also called an antisense box, the guide sequence base pairs with the target, forming an RNA:RNA duplex. The nucleotide targeted for 2’-O-ribose methylation is usually base paired with the fifth residue upstream from either the box D, or D’ [2, 19] (see **Fig 1A**). We have demonstrated that these methylation guide sequences in snoRNAs are not required for target gene knock-down by snoMEN vectors [7]. In the case of box H/ACA snoRNAs, which catalyse psuedouridine formation by the conserved snoRNP protein dyskerin, the hairpins contain bulges, or recognition loops, that form complex pseudo-knots with the target RNA, where the target uridine is the first unpaired base [2, 3] (see **Fig 1A**). The position of the substrate uridine always resides 14–16 nucleotides upstream of either the H box, or of the ACA box. Compared with the snoRNA guide sequences targeting pseudouridylation, the M box is located at a single stranded region and is not predicted to form a recognition loop. Taken together, the current evidence suggests that the mechanism of targeted gene knock-down by snoMEN is independent of the guide sequences used by box C/D and box H/ACA snoRNAs to target 2’-O-ribose methylation and pseudouridylation, respectively.

We have reported that certain mammalian miRNA precursors appear closely related to snoRNAs, with a subset of miRNA precursors and snoRNAs showing similarities in their primary and secondary structures (see **Fig 1**). Indeed, we have also identified known miRNAs that are encoded within snoRNA-like molecules [20–23] (**Fig 1A** and **Fig B** in **S1 File**). Furthermore, other groups have investigated known snoRNAs that are further processed into miRNA-like fragments [24–27], supporting a fundamental relationship between snoRNAs and miRNAs, which we speculate may be of relevance to the evolutionary origin of some types of miRNAs. These observations raise the possibility that snoMEN are actually miRNA precursors, which predicts that the mechanism of knock-down by snoMEN involves the previously characterised miRNA biogenesis pathway. Our previous analyses have indicated that snoMEN-mediated knock-down occurs via a mechanism that may involve the Ago2 and/or Upf1 proteins, but is independent of Ago1 [9]. In addition, we observe that snoMEN RNAs share characteristic features seen for endogenous snoRNAs, including steady state accumulation in nucleoli, inactivation by mutations in snoRNA core motifs and binding with conserved snoRNP proteins. This suggests that targeted gene knock-down by snoMEN RNAs may involve a mechansim distinct from the canonical miRNA/shRNA/siRNA maturation pathways, even if some common components may be involved.

In this study we have identified the U47 snoRNA as a superior snoMEN vector backbone. Data from FP reporter assays showed that the knock-down efficiency of box C/D type U47snoMEN and U77snoMEN was higher than obtained with the box H/ACA type ACA16snoMEN and miR-566snoMEN, for both mRNA and protein levels (**Figs 4** and **5**). As the majority of box H/ACA snoRNAs are longer than box C/D snoRNAs and also form more complex secondary and tertiary structures (For example see **Fig 1A**), it is possible that the stability of shorter snoRNAs, i.e. box C/D type snoMEN, are more stable than box H/ACA type snoMEN. Based upon its short size and knock-down efficiency, we concentrated here on developing new snoMEN vectors based on the U47 snoRNA backbone.

We previously demonstrated the establishment of human protein replacement stable cell lines using the original HBII-180C snoMEN vectors [9] (**Fig 8B**). However, the efficiency of gene knock-in obtained was relatively low, e.g. FP-tagged versions of proteins replaced ~30% of the corresponding endogenous protein level (**Fig 9**). To improve the knock-in efficiency, we have characterised here vectors using the new U47 snoRNA backbone and also have modified the vector design to improve exogenous protein expression by locating the cDNA coding sequence downstream (3’) of the multiplex snoMEN sequences in the knock-in transcript. We observe that this version of the snoMEN vector resulted in increased (~5 fold higher) expression of exogenous, knock-in FP-tagged proteins (**Fig 9**). This may largely result from avoiding activation of the NMD pathway as a result of locating introns in the transcript downstream of the stop codon in the cDNA sequence [28–30] (**Fig 8A** and **8C**). In combination with the improved knock-down efficiency of the targeted endogenous protein resulting from the new U47 snoRNA backbone, the higher exogenous protein expression from the vector thus provides a substantial improvement in knock-in efficiency.

Using the improved vector design allowed us to establish new human stable cell lines, including cells with knock-in of GFP–HIF-1α. Previous work has shown that it is very challenging to establish stable cell lines expressing knock-in of GFP–HIF-1α using alternative technological approaches (our unpublished observations). Characterising these protein replacement stable cell lines using a combination of assays, including fluorescence microscopy and quantitative mass spectrometry, showed that the 47snoMEN-PR vectors generated efficient knock-in of targeted proteins, with little or no reduction in either cell growth, or viability and with minimal off-target effects on the expression levels of non-targeted proteins, as judged by quantitative proteomics analysis. We conclude, therefore, that the 47snoMEN vectors expand the repertoire of technologies available for manipulating gene expression in mammalian cells and can provide new opportunities for overcoming current limitations in gene expression studies, as exemplified here for GFP–HIF-1α. We foresee further development of 47snoMEN vectors to expand their utility and applications, e.g. incorporating inducible promoters and delivery using virus-based vectors. With the demonstrated improvements in knock-in efficiency, we anticipate future applications for 47snoMEN vectors in basic gene expression research, in drug screening and target validation studies and possibly also for gene therapy.

## Methods

### Secondary structure analysis

All RNA secondary structures were predicted by RNAstructure 4.6 and annotated using RnaViz 2.0.

### Cell culture

HeLa and U2OS cells were provided from EMBL and ATCC, respectively. HeLa and U2OS cells were maintained in Dulbecco’s modified Eagle’s medium (DMEM) supplemented with 10% fetal bovine serum (FBS).

### Plasmid construction and transfections

The sequence spanning exon 2 to exon 3 of the C19orf48 gene and the sequence spanning exon 5 to exon 6 of the TBRG4 (transforming growth factor beta regulator 4) gene were inserted 3’ of the CMV promoter in the pcDNA3.1 mammalian expression plasmid (Invitrogen), creating U47/U77 box C/D type snoMEN and ACA16/miR-566 box H/ACA type snoMEN expression vectors. SnoMEN and mutant snoMEN derivatives of each snoRNA were established from the respective wild type snoRNA expression mini gene constructs by site directed mutagenesis. The plasmids were transfected into either HeLa, or U2OS cells, using “effectine” transfection regent (QIAGEN).

### Fluorescent *in situ* hybridization (FISH)

FISH procedure was performed as previously described [http://www.singerlab.org/protocols]. HeLa cells were transfected with a plasmid vector containing the snoMEN mini gene expressed from the CMV promoter. The cells were fixed with 4% paraformaldehyde after pre-permeabilization with 1% tritonX-100. After 70% ethanol treatment, a Cy-3 labeled snoMEN specific oligonucleotide probe (CM1: 5’-GCAGCACGACUUCUUCAAGUC-3’, CM2: 5’-GCAGAAGAACGGCAUCAAGGU-3’ CM3: 5’-CAGCCACAACGUCUAUAUCAU-3’) was hybridized with Fluorescein labelled U3 specific oligonucleotide probe (5’-ACCACUCAGACCGCGUUCUCUCCC-3’) as a nucleolar marker, using standard procedures. The fluorescence signal was imaged using a Deltavision Spectris fluorescence microscope (Applied Precision). The specific probes for each of the snoRNAs (U47: 5’-UGGAACGGUUUUACAGUGAUAUC-3’, U77: 5’-ACAUCUCUUCAUGAUUAAAUcugcug-3’, ACA16: 5’-ACCGUCAAGGAAAACUGUCACUCUGGGC-3’, miR-566: 5’-GUUGGGAUCACAGGCGCCC-3’) were also labelled with Cy-3.

### Microscopy imaging and antibodies

All cell images were recorded using the DeltaVision Spectris fluorescence microscope (Applied Precision). Live cell images were prepared as previously described (http://www.lamondlab.com/f7protocols.htm). Cells were imaged using a 60x (NA 1.4) Plan Apochromat objective. Twelve optical sections separated by 0.5 μm were recorded for each field and each exposure (SoftWoRx image processing software, Applied Precision). Primary antibodies against SMN1 (BD Transduction Laboratories), fibrillarin (72b9) [31], Dyskerin (Sigma-Aldrich) and coilin (5P10) [32] were used for immunostaining. Primary antibodies against HIF-1α (610958, BD Biosciences), HIF-1β (3718, Cell Signaling), CAIX (NB100-417, Novus Biologicals), PHD2 (Bethyl A300-322A), BNIP3 (ab10433, Abcam), PHD3 (A300-327A, Bethyl labs) and Actin (3700, Cell Signaling) were used for immuno blotting analysis.

### Immunoprecipitation and RT-PCR

Immunoprecipitations were prepared as previously described [20, 33–36]. Nuclear lysates were prepared from HeLa^YFP-Fibrillarin^ [33], HeLa^GFP-Dyskerin^ [20] and HeLa^GFP^ [20, 33] stable cell lines. Purified nuclei were resuspended in RIPA buffer to solubilize proteins. Fluorescent proteins were immunoprecipitated using an anti-GFP monoclonal antibody (GFP–TRAP_A, Chromotek), as previously described [20, 33]. Samples were divided in two and for Input samples RNA was isolated from one half of each nuclear lysate. RNA was isolated by the TRIzol method with DNase I treatment, according to manufacturer’s instruction (Invitrogen). RT-PCR was performed to detect immunoprecipitated RNAs. Reverse transcription and PCR were performed with the following gene-specific primers,

U3: 5’-AGAGGTAGCGTTTTCTCCTGAGCG-3’ and 5’-ACCACTCAGACCGCGTTCTC-3’,

E2: 5’-GGAGTTGAGGCTACTGACTGGC-3’ and 5’-CCACTCATTGGGCCAGAGACCC-3’,

U47snoMEN: 5’-TAATGATTCTGCCAAATGAATCACC-3’ and 5’-ACCTCAGAATCAAAATGGAACGGC-3’,

U77snoMEN: 5’- AGATACTATGATGGTCACCTTGATGC-3’ and 5’-

AGATACTATGATGGTTGCATAGTTCAG-3’,

ACA16snoMEN: 5’-TGCTTCCGCATAGCTGCTGTGG-3’ and 5’-

CAAGCAGAAGCAGTTACAACAAACAG-3’

miR-566snoMEN: 5’-ACCTTGATGCCGTTCTTCTGC-3’ and 5’-GGTTCAAGCGATTCTGCTGCCC-3’

HBII-180C snoMEN: 5’-ACCTTGATGCCGTTCTTCTGC-3’ and 5’-CTCAGACCCCCAGGTGTCAA-3’,

using the one-step RT-PCR kit (QIAGEN). To decide linearity of cycles, we carried out real time PCR using the QuantiFast SYBR Green one-step qRT-PCR Kit (QIAGEN) and Light Cycler 480II (Roche). The same amount of RNA for input and immunoprecipitated RNA (IP) was used as templates for RT-PCR reactions. Each experiment was repeated three times independently.

### Quantitative RT-PCR (qRT-PCR)

The qRT-PCR was performed to measure GFP-SMN1 expression level after snoMEN plasmid transfections. Equal amounts of total RNA from HeLa cells, extracted following snoMEN transfections, were used for qRT-PCR reactions. The GFP-SMN1 mRNA expression ratio between control and snoMEN transfection measured from four independent experiments. GFP specific primers (5’-GACCACTACCAGCAGAACACCC-3’ and 5’-TGTGATCGCGCTTCTCGTTGGG-3’) U3 mRNA specific primers (5’-AGAGGTAGCGTTTTCTCCTGAGCG-3’ and 5’-ACCACTCAGACCGCGTTCTC-3’), as a loading control, were used for amplification.

### Quantification analysis of blotting images

All signal intensities of Western blotting images were analysed using imaging software (Image Gauge v4.21; Fujifilm) with the default manufacturer’s procedure. Briefly, the same size of pixel area was selected and signal intensity calculated by subtraction of the background signal. Each signal was normalised with reference to standard control signals, e.g. tubulin, and a signal to control ratio was calculated.

### Proliferation/cytotoxicity assay

Proliferation/cytotoxicity assays were performed using alamarBlue (AbD Serotec) as described by the supplier. Fluorescence was measured using a CLARIOstar plate reader (BMG LABTECH).

### Stable isotope-labelling of cellular proteins and mass spectrometry sample preparations

SILAC experiments were performed as previously described [34–40]. Cells were grown for at least six cell divisions in L-arginine-, or L-arginine ^13^C_6_^15^N_4_-labelling media before analysis. Cells were resuspended in 8% Urea—0.1M TEAB buffer to solubilize proteins. Lysates from each cell were mixed in a 1:1 ratio based on total protein concentration and sonicated to fragment genomic DNA. Proteins were reduced with TCEP (25 mM in denaturing urea buffer) and alkylated with iodoacetamide. Lysates were diluted with digest buffer (100 mM Tris pH 8.0 + 1 mM CaCl_2_) to reach 4 M urea, and then digested with 1:50 Lys-C (Wako Chemicals, Japan). The digest was diluted with digest buffer to reach 0.8 M urea and digested with trypsin (Promega; 1:50). The digests were then desalted using SepPak-C18 SPE cartridges, dried and resuspended in 80% Acetonitrile, 0.1% TFA. Peptides were separated using a Dionex Ultimate 3000 HPLC system equipped with a HILIC (hydrophilic interaction liquid chromatography) column, using a similar protocol to the hSAX method described previously [41]. The peptide fractions were desalted using SepPak-C18 SPE plates and then resuspended in 5% formic acid for LC-MS/MS analysis.

### Mass spectrometry and data analysis

For Trypsin+Lys-C double digests, peptide chromatography was performed using a Dionex RSLCnano HPLC. Peptides were injected directly onto a 75 μm × 50 cm PepMap-C18 column using the following mobile phases: 2% acetonitrile +0.1% formic acid (Solvent A) and 80% acetonitrile +0.1% formic acid (Solvent B). The linear gradient began with 5% A to 35% B over 220 min with a constant flow rate of 200 nl/min. The peptide eluent flowed into a nanoelectrospray emitter at the front end of a Q-Exactive (quadrupole Orbitrap) mass spectrometer (Thermo Fisher). A typical ‘Top10’ acquisition method was used and an in-house developed software (Peptracker [42]) was used to evaluate peptide identifications and abundance ratios. The mass spectrometry proteomics data have been deposited to the ProteomeXchange Consortium via the PRIDE [43] partner repository with the dataset identifier PXD003622.

### Drug treatments

MG132 was obtained from Merck Millipore and used at 20μM final concentration for 3 hours, Desferroxamine (DFX) was obtained from Sigma and used at the final concentration of 200μM for 24 hours, Dimethyloxalylglycine (DMOG) was obtained from Merck Millipore and used at a final concentration of 1mM for 24 hours.

### Hypoxia treatment and 3D culture analysis

Cells were incubated at 1% O_2_ in an *in vivo* 300 hypoxia workstation (Ruskin, UK). Cells were lysed for protein extracts in the workstation to avoid reoxygenation. For 3-D culture, Lipidure plates (NOF America Corporation #51011610) were used for spheroid formation due to their improved reproducibility over conventional methods and the formation of a single, central spheroid. Lipidure plates are pre-coated with phosphorylcholine to supress cell adhesion. 200μl of 5x10^4^ live cells per mL were seeded per well and spheroids allowed to form overnight. Growth of formed spheroids was documented by light microscopy

## Supporting Information

S1 Data Supporting Table of *in silico* analysis. (DOCX)

S2 Data Supporting Table of SILAC analysis. (XLSX)

S1 File Supporting information. (PDF)
