# Supplementary material for: Enhanced snoMEN Vectors Facilitate Establishment of GFP–HIF-1α Protein Replacement Human Cell Lines
Source: PLoS One. 2016 Apr 29;11(4):e0154759. doi: 10.1371/journal.pone.0154759 (PMC4851398; doi:10.1371/journal.pone.0154759)
Supplement: S1 Data — (DOCX) [file pone.0154759.s001.docx]

**Supplementary Data File 1**

To investigate the number of potential complementary snoRNA targets in transcripts, pre-mRNA sequences were scanned for regions of complementarity to snoRNAs. snoRNA sequences were extracted from snoRNAbase [[1](#_ENREF_1)] and all hg38 transcript sequences (including all introns and exons) were extracted from the RefSeq Genes (refGene) table in the UCSC Table Browser [[2](#_ENREF_2)].

RNA-RNA duplex interactions were predicted using RNAplex [[3](#_ENREF_3)] with the following parameters:

-Energy threshold for an interaction to be returned of -0.2 kcal/(mol·nt) (-e -20)

-Fixed extension penalty of 0.5 kcal/(mol·nt) (-c 50)

-Window size of 1 for the best candidate for the backtracking step (-z 1)

The quality of each predicted interaction was evaluated using an in house script to ensure the presence of a minimum number of matches in a window of n nucleotides as a threshold to keep the predicted interactions. Here we kept only the predicted interactions having at least 21 matches in a window of 22 nucleotides, which corresponds to hits of the same quality as the previously validated HBII-180C hits [[4](#_ENREF_4),[5](#_ENREF_5)].

For box C/D snoRNAs, only hits involving a region of complementarity of 21 matches in a window of 22 nucleotides between the boxes C and D were counted.

The number of such snoRNA complementary hits in the human pre-mRNA transcriptome is shown for each snoRNA in the table below. The snoRNA region most often found complementary to a target is indicated in the column ‘Predominant M-box’ for each snoRNA, and the position of this region in the snoRNA is indicated in the column ‘M-box position in snoRNA’.

**References**

1. Lestrade L, Weber MJ (2006) snoRNA-LBME-db, a comprehensive database of human H/ACA and C/D box snoRNAs. Nucleic Acids Res 34: D158-162.

2. Kuhn RM, Haussler D, Kent WJ (2013) The UCSC genome browser and associated tools. Brief Bioinform 14: 144-161.

3. Tafer H, Hofacker IL (2008) RNAplex: a fast tool for RNA-RNA interaction search. Bioinformatics 24: 2657-2663.

4. Scott MS, Ono M, Yamada K, Endo A, Barton GJ, et al. (2012) Human box C/D snoRNA processing conservation across multiple cell types. Nucleic Acids Res 40: 3676-3688.

5. Ono M, Yamada K, Avolio F, Scott MS, van Koningsbruggen S, et al. (2010) Analysis of human small nucleolar RNAs (snoRNA) and the development of snoRNA modulator of gene expression vectors. Mol Biol Cell 21: 1569-1584.

**Supplementary table 1**: Number of predicted complementary targets in pre-mRNA, per snoRNA

| **snoRNA name** | **snoRNA type** | **Number of hits** | **Predominant M-box** | **M-box position in snoRNA** |
| --- | --- | --- | --- | --- |
| 14q(0) | C/D | 5 | UCAUGAUUAAUCCAGUUCUGCA | 36-57 |
| 14q(I-1) | C/D | 1 | GAAUAGUUCUGUGGCAUAUGAA | 12-33 |
| 14q(I-2) | C/D | 10 | AGCUGUAGGAA | 22-32 |
| 14q(I-3) | C/D | 12 | UGGGGUGUCUGAA | 20-32 |
| 14q(I-4) | C/D | 16 | GGGGUAUCUGA | 21-31 |
| 14q(I-5) | C/D | 29 | GGUGGAGGUGUCUG | 19-32 |
| 14q(I-6) | C/D | 1 | UGAAUAUCAUGGGGUUUCUGAAA | 11-33 |
| 14q(I-7) | C/D | 7 | GAGUAUGCGUGGGGCAUCUGA | 12-32 |
| 14q(I-8) | C/D | 5 | GAUUGGAGGGUGUCUGAAUCA | 14-34 |
| 14q(I-9) | C/D | 39 | GGGUGUCUGAA | 22-32 |
| 14q(II-1) | C/D | 20 | GAGUCAUUGAC | 29-39 |
| 14q(II-10) | C/D | 4 | AGUGUAUGAGU | 22-32 |
| 14q(II-11) | C/D | 89 | GGUGUGUGAGU | 22-32 |
| 14q(II-12) | C/D | 7 | CCGGCGUAUGAGUC | 20-33 |
| 14q(II-13) | C/D | 24 | CAUAUGAGUCAUA | 24-36 |
| 14q(II-14) | C/D | 39 | UGAGUGUUGGG | 28-38 |
| 14q(II-15) | C/D | 11 | UGGCGUAUGAGUC | 21-33 |
| 14q(II-16) | C/D | 15 | AGUCACGCAUA | 25-35 |
| 14q(II-17) | C/D | 47 | GGCAUUGGAGU | 22-32 |
| 14q(II-18) | C/D | 12 | GGUGUAUGAGUC | 22-33 |
| 14q(II-19) | C/D | 22 | UGAAUCUUGGA | 28-38 |
| 14q(II-2) | C/D | 100 | AUGAGUCAGUG | 27-37 |
| 14q(II-20) | C/D | 17 | GAGUCAUCUAC | 29-39 |
| 14q(II-21) | C/D | 16 | GGUGGCGUAUGAGUC | 19-33 |
| 14q(II-22) | C/D | 19 | GGUGGCGUAUGAGUCAUA | 19-36 |
| 14q(II-23) | C/D | 11 | GCGUAUGAGUCA | 23-34 |
| 14q(II-24) | C/D | 16 | AUGGCAUAUGA | 20-30 |
| 14q(II-25) | C/D | 22 | GUGGCGUAUGAGUCU | 20-34 |
| 14q(II-26) | C/D | 29 | GUGGAGUAUGAG | 20-31 |
| 14q(II-27) | C/D | 8 | CUGGUGUCGUGUGAG | 17-31 |
| 14q(II-28) | C/D | 23 | GGUGGCGUAUGAGUC | 19-33 |
| 14q(II-29) | C/D | 19 | CAUGAGUCAUA | 24-34 |
| 14q(II-3) | C/D | 59 | GGCGUUUGAGU | 22-32 |
| 14q(II-30) | C/D | 66 | AUGGGUCUUCG | 27-37 |
| 14q(II-31) | C/D | 115 | UGUGAGUCAUG | 26-36 |
| 14q(II-4) | C/D | 55 | AGAAGUCAAGG | 27-37 |
| 14q(II-5) | C/D | 21 | GGCCUAUGAGUC | 22-33 |
| 14q(II-6) | C/D | 2 | GGUGAAUAUAU | 40-50 |
| 14q(II-7) | C/D | 30 | AUAUUUAGUCA | 25-35 |
| 14q(II-8) | C/D | 28 | GGCUUAUGAGU | 22-32 |
| 14q(II-9) | C/D | 12 | GUGGCGUAUGAGUC | 20-33 |
| ACA1 | H/ACA | 209 | CCAUCACAGUA | 81-91 |
| ACA10 | H/ACA | 371 | GCUUGGCGUGU | 39-49 |
| ACA13 | H/ACA | 319 | CCUUUUCCUGC | 76-86 |
| ACA14a | H/ACA | 262 | CUCCUGUGGCU | 96-106 |
| ACA14b | H/ACA | 241 | GCUGCGCUUCUC | 88-99 |
| ACA15 | H/ACA | 90 | CAGUCUUCCCU | 58-68 |
| ACA16 | H/ACA | 238 | GCUUCCGCAUA | 24-34 |
| ACA17 | H/ACA | 572 | GCUGUGGCUGC | 21-31 |
| ACA18 | H/ACA | 362 | GGCUUUUCCUG | 20-30 |
| ACA19 | H/ACA | 33 | GCUGCCAGUCUCCU | 104-117 |
| ACA20 | H/ACA | 176 | CAUCUCUUCUG | 105-115 |
| ACA21 | H/ACA | 64 | GGUGAAGUGGC | 84-94 |
| ACA22 | H/ACA | 483 | GCCUGCUCUUC | 64-74 |
| ACA23 | H/ACA | 169 | UGGUAGCAGUG | 39-49 |
| ACA24 | H/ACA | 241 | GGCAGUCUCCC | 30-40 |
| ACA25 | H/ACA | 162 | CUGUGAAACCC | 27-37 |
| ACA27 | H/ACA | 308 | GUGUUUGUCUG | 93-103 |
| ACA28 | H/ACA | 195 | GUUCUCUCUCC | 82-92 |
| ACA29 | H/ACA | 390 | GGGUUUUUUCA | 92-102 |
| ACA2a | H/ACA | 556 | GUGGUCUGUUG | 74-84 |
| ACA2b | H/ACA | 168 | GCUGUUGGUUU | 33-43 |
| ACA3 | H/ACA | 280 | CUGUGGGCCUUA | 86-97 |
| ACA30 | H/ACA | 318 | GCAGUGGGGCC | 27-37 |
| ACA31 | H/ACA | 549 | GUCUGUCUUUG | 87-97 |
| ACA32 | H/ACA | 300 | UCUAUCUGCUA | 78-88 |
| ACA3-2 | H/ACA | 201 | GGGGGCAGUCG | 87-97 |
| ACA33 | H/ACA | 95 | GGUCCCUGAGA | 107-117 |
| ACA34 | H/ACA | 635 | UCUGUUGGCUG | 78-88 |
| ACA36 | H/ACA | 230 | GGGCAGCUUCC | 24-34 |
| ACA36B | H/ACA | 217 | GGUAGCUUCCC | 25-35 |
| ACA37 | H/ACA | 399 | GCUGUGGGGCC | 27-37 |
| ACA38 | H/ACA | 455 | GUUCCCUGUCU | 24-34 |
| ACA39 | H/ACA | 442 | CUCCUGUCCCA | 108-118 |
| ACA4 | H/ACA | 142 | UGGGGGGCAGG | 18-28 |
| ACA40 | H/ACA | 62 | CCCAGAACUCA | 75-85 |
| ACA41 | H/ACA | 254 | CUGCAGCUGUU | 16-26 |
| ACA42 | H/ACA | 525 | UCUCUGUGGGC | 25-35 |
| ACA43 | H/ACA | 415 | GCUGUGCCUGU | 38-48 |
| ACA44 | H/ACA | 232 | CUGUGGCUGGU | 17-27 |
| ACA46 | H/ACA | 332 | UUGCACAGCUG | 80-90 |
| ACA48 | H/ACA | 549 | GCCCAUGCCUG | 103-113 |
| ACA49 | H/ACA | 307 | UACUCCAGGGA | 42726 |
| ACA5 | H/ACA | 425 | GUUCCUGUGUC | 89-99 |
| ACA50 | H/ACA | 139 | CUCUUCAGCUC | 89-99 |
| ACA51 | H/ACA | 696 | GCCCUGUGCCU | 93-103 |
| ACA52 | H/ACA | 410 | GGGCUCCAGUG | 93-103 |
| ACA53 | H/ACA | 311 | UUGUGGUUCCA | 70-80 |
| ACA54 | H/ACA | 318 | GCUUUCAGUUU | 94-104 |
| ACA55 | H/ACA | 686 | UUGCUGCCUCG | 24-34 |
| ACA56 | H/ACA | 594 | UCUGUCCAGCG | 79-89 |
| ACA58 | H/ACA | 363 | AGGCAGGGGGG | 39-49 |
| ACA59 | H/ACA | 136 | CAGCUGGCCCA | 35-45 |
| ACA59B | H/ACA | 136 | CAGCUGGCCCA | 35-45 |
| ACA5b | H/ACA | 574 | UUACUGUGUCC | 88-98 |
| ACA5c | H/ACA | 310 | GCCCUGGGUCAC | 85-96 |
| ACA6 | H/ACA | 316 | GAGGCCAGUCU | 24-34 |
| ACA60 | H/ACA | 248 | GCCUGUGCUCUG | 28-39 |
| ACA61 | H/ACA | 176 | CUUUCCCAUCG | 13-23 |
| ACA62 | H/ACA | 213 | CUGGCCUCCCG | 91-101 |
| ACA63 | H/ACA | 377 | UGGCUGCCUCA | 77-87 |
| ACA64 | H/ACA | 471 | GGCCGUGGCCG | 26-36 |
| ACA65 | H/ACA | 399 | UGUUGCCUUCA | 16-26 |
| ACA67 | H/ACA | 289 | CCUCUGUUCCC | 91-101 |
| ACA67B | H/ACA | 222 | CUCUGUGCCCG | 92-102 |
| ACA7 | H/ACA | 382 | CAUUCCCAGCU | 88-98 |
| ACA7B | H/ACA | 367 | CAUUCCCAGCU | 88-98 |
| ACA8 | H/ACA | 371 | GUUUUCUUGGC | 89-99 |
| ACA9 | H/ACA | 404 | CUUGCCCUGCU | 86-96 |
| E2 | H/ACA | 187 | AGUUGAGGCUA | 22-32 |
| E3 | H/ACA | 169 | GCAGCCAGGGA | 105-115 |
| HBI-115 | H/ACA | 356 | UUGGAGGGGCA | 105-115 |
| HBI-36 | H/ACA | 227 | GCUACUCCUGC | 99-109 |
| HBI-43 | C/D | 299 | AGGGACGGGGC | 180-190 |
| HBI-6 | H/ACA | 475 | CCCAGUGCUUU | 18-28 |
| HBI-61 | H/ACA | 119 | GGGGUGGACCU | 119-129 |
| HBII-108 | C/D | 12 | GUCUGAUAGUC | 24-34 |
| HBII-108B | C/D | 0 | CCAACUCUGAUUUCA | 62-76 |
| HBII-115 | C/D | 497 | CCUGGGCUGGG | 67-77 |
| HBII-13 | C/D | 56 | UACUGAGCAUG | 21-31 |
| HBII-135 | C/D | 49 | UACCGGCAGAU | 31-41 |
| HBII-142 | C/D | 96 | CGUGUCUGGGC | 28-38 |
| HBII-166 | C/D | 127 | GAGCCAUGGUA | 28-38 |
| HBII-180A | C/D | 40 | CCCCGGGACCU | 57-67 |
| HBII-180B | C/D | 100 | GACUGCCCCUG | 33-43 |
| HBII-180C | C/D | 53 | CUGAUCACCCCUG | 31-43 |
| HBII-202 | C/D | 34 | CUGUACGGCCU | 25-35 |
| HBII-210 | C/D | 48 | ACUGUGCUGAGU | 36-47 |
| HBII-234 | C/D | 19 | GAAUCUAAGUGA | 35-46 |
| HBII-239 | C/D | 37 | GGCUAAGUGUC | 36-46 |
| HBII-240 | C/D | 20 | UGCAGUAUUGAU | 44-55 |
| HBII-251 | C/D | 42 | GCCCUCACUUA | 24-34 |
| HBII-276 | C/D | 93 | UACCCAGCUGA | 31-41 |
| HBII-289 | C/D | 170 | GCAGUUUGCUC | 47-57 |
| HBII-295 | C/D | 27 | AGGGCAGAUUCUGAGGU | 32-48 |
| HBII-296A | C/D | 43 | UCUCAGUGAUG | 51-61 |
| HBII-296B | C/D | 33 | CCAGUGAUGCA | 50-60 |
| HBII-316 | C/D | 72 | CUCACUGAGAG | 36-46 |
| HBII-336 | C/D | 22 | UGCUUCUGCUG | 34-44 |
| HBII-419 | C/D | 55 | UCCAUUGCUGA | 25-35 |
| HBII-420 | C/D | 8 | CAUGGAUGAGAAAU | 36-49 |
| HBII-429 | C/D | 37 | ACUGAACUGCC | 29-39 |
| HBII-436 | C/D | 6 | CAGGACCUUGUCUGAA | 14-29 |
| HBII-437 | C/D | 31 | CUUUCCAUUCCUG | 40-52 |
| HBII-438A | C/D | 75 | GCUGAGGGACU | 31-41 |
| HBII-438B | C/D | 75 | GCUGAGGGACU | 31-41 |
| HBII-52-1 | C/D | 12 | CCUGAAGAGAGG | 27-38 |
| HBII-52-10 | C/D | 4 | GUCUGAAGAGAG | 25-36 |
| HBII-52-11 | C/D | 30 | CCUGAAGAGAG | 27-37 |
| HBII-52-12 | C/D | 30 | CCUGAAGAGAG | 27-37 |
| HBII-52-13 | C/D | 11 | CCUGAAGAGAGG | 27-38 |
| HBII-52-14 | C/D | 2 | GUCUGAAGAGAG | 25-36 |
| HBII-52-15 | C/D | 6 | UUCUGAAGAGA | 25-35 |
| HBII-52-16 | C/D | 12 | CCUGAAGAGAGG | 27-38 |
| HBII-52-17 | C/D | 31 | CCUGAAGAGAG | 27-37 |
| HBII-52-18 | C/D | 31 | CCUGAAGAGAG | 27-37 |
| HBII-52-19 | C/D | 31 | CCUGAAGAGAG | 27-37 |
| HBII-52-2 | C/D | 37 | CUGUUUUCUUGAAG | 20-33 |
| HBII-52-20 | C/D | 31 | CCUGAAGAGAG | 27-37 |
| HBII-52-21 | C/D | 1 | UCUGAAGAGAGGUGAUGACU | 26-45 |
| HBII-52-22 | C/D | 44 | CCUGAAGAGAG | 27-37 |
| HBII-52-23 | C/D | 13 | UGUUGAAGAGA | 26-36 |
| HBII-52-25 | C/D | 29 | CCUGAAGAGAG | 27-37 |
| HBII-52-26 | C/D | 30 | CCUGAAGAGAG | 27-37 |
| HBII-52-29 | C/D | 30 | CCUGAAGAGAG | 27-37 |
| HBII-52-3 | C/D | 29 | CCUGAAGAGAG | 27-37 |
| HBII-52-30 | C/D | 2 | GUUCUGAAGAGAGG | 25-38 |
| HBII-52-31 | C/D | 22 | GUCCUGAAGAA | 25-35 |
| HBII-52-32 | C/D | 24 | GCCCUGAAGAG | 25-35 |
| HBII-52-33 | C/D | 7 | CCUGAAGAGCGG | 27-38 |
| HBII-52-34 | C/D | 3 | UCUGAAGAGAGGUGAUGACU | 27-46 |
| HBII-52-35 | C/D | 8 | UAUCUUGAAGA | 24-34 |
| HBII-52-36 | C/D | 30 | CCUGAAGAGAG | 27-37 |
| HBII-52-37 | C/D | 25 | AGGUGAUGACU | 36-46 |
| HBII-52-38 | C/D | 16 | UCCUGAAGAGA | 26-36 |
| HBII-52-39 | C/D | 27 | CCUGAAGAGAGGUG | 27-40 |
| HBII-52-4 | C/D | 4 | UCUGAAGAGAG | 27-37 |
| HBII-52-40 | C/D | 11 | UCCUGAAGAGAG | 26-37 |
| HBII-52-41 | C/D | 37 | CCUGAAGAGAG | 27-37 |
| HBII-52-42 | C/D | 3 | UCUGAAGAGAGGUGAUGACU | 27-46 |
| HBII-52-43 | C/D | 30 | CCUGAAGAGAG | 27-37 |
| HBII-52-44 | C/D | 13 | CCUGAAGAGCG | 27-37 |
| HBII-52-48 | C/D | 3 | GAGAAAUGAUGACGUAA | 27-43 |
| HBII-52-5 | C/D | 30 | CCUGAAGAGAG | 27-37 |
| HBII-52-6 | C/D | 3 | UCUGAAGAGAGGUGAUGACU | 27-46 |
| HBII-52-7 | C/D | 24 | CCUGAAGAGAGG | 24-35 |
| HBII-52-8 | C/D | 4 | CUGAAGAGAGA | 28-38 |
| HBII-52-9 | C/D | 30 | CCUGAAGAGAG | 27-37 |
| HBII-55 | C/D | 20 | GCAAUCACUGA | 43-53 |
| HBII-82 | C/D | 335 | UUUUUCUCUGG | 49-59 |
| HBII-82B | C/D | 24 | GUCUCUUCUCUG | 27-38 |
| HBII-85-1 | C/D | 7 | AAACAUUCCUUGGA | 25-38 |
| HBII-85-10 | C/D | 24 | CAGUACCAUCAUCCU | 72-86 |
| HBII-85-11 | C/D | 111 | GGUUCCUUGGA | 26-36 |
| HBII-85-12 | C/D | 37 | UAUACAUUCCU | 22-32 |
| HBII-85-13 | C/D | 66 | CAUGCAUUCCU | 22-32 |
| HBII-85-14 | C/D | 37 | UAUACAUUCCU | 22-32 |
| HBII-85-15 | C/D | 34 | UAUACAUUCCU | 22-32 |
| HBII-85-16 | C/D | 41 | GCAUUCCUUGG | 25-35 |
| HBII-85-17 | C/D | 39 | UAUACAUUCCU | 22-32 |
| HBII-85-18 | C/D | 40 | ACAUUCCUUGG | 25-35 |
| HBII-85-19 | C/D | 39 | UAUACAUUCCU | 22-32 |
| HBII-85-2 | C/D | 8 | CAUACCGUCAUUCUCAUCG | 65-83 |
| HBII-85-20 | C/D | 37 | UAUACAUUCCU | 22-32 |
| HBII-85-21 | C/D | 55 | UAUACAUUCCU | 22-32 |
| HBII-85-22 | C/D | 41 | UACAUUCCUUGG | 24-35 |
| HBII-85-23 | C/D | 26 | GCAUUCCUUGG | 25-35 |
| HBII-85-24 | C/D | 24 | CAUUCCUUGGA | 26-36 |
| HBII-85-25 | C/D | 19 | CUUCUGUGCCA | 63-73 |
| HBII-85-26 | C/D | 13 | CAUUUCUGUGC | 65-75 |
| HBII-85-27 | C/D | 6 | CUUCUGUACCACU | 63-75 |
| HBII-85-28 | C/D | 53 | UGAGCAAACCA | 52-62 |
| HBII-85-29 | C/D | 0 | - | - |
| HBII-85-3 | C/D | 13 | CAUACCGUCGUUCUCA | 65-80 |
| HBII-85-4 | C/D | 8 | CAUACCGUCGUUCUCAGCG | 66-84 |
| HBII-85-5 | C/D | 15 | CAUACCGUCGUUCUCA | 65-80 |
| HBII-85-6 | C/D | 11 | UCAUACCGUCA | 65-75 |
| HBII-85-7 | C/D | 15 | CAUACCGUCGUUCUCA | 65-80 |
| HBII-85-8 | C/D | 16 | UCAUACCGUCG | 64-74 |
| HBII-85-9 | C/D | 13 | CAUACCGUCGUUCUCA | 65-80 |
| HBII-95 | C/D | 101 | CCUGAUUUCCU | 28-38 |
| HBII-95B | C/D | 31 | GGGUGUUCAGU | 56-66 |
| HBII-99 | C/D | 82 | CCCAUCAGAUCG | 30-41 |
| mgh18S-121 | C/D | 2 | GUCUGAACAAAGUGAUUG | 32-49 |
| mgh28S-2409 | C/D | 10 | ACUGCUGAAUG | 26-36 |
| mgh28S-2411 | C/D | 49 | CUGAAGGGGCU | 34-44 |
| mgU6-47 | C/D | 291 | GCUUCACUGUUG | 43-54 |
| mgU6-53 | C/D | 325 | GCCCUGGUUUG | 52-62 |
| mgU6-53B | C/D | 109 | GGUAGGGAGUU | 38-48 |
| mgU6-77 | C/D | 547 | CAGGGCUGUUG | 78-88 |
| SNORA11B | H/ACA | 212 | GAGCAGGGGGC | 42726 |
| SNORA11C | H/ACA | 273 | GAGCAGGGGGC | 42726 |
| SNORA11D | H/ACA | 307 | AGCAGGGGGCC | 13-23 |
| SNORA11E | H/ACA | 264 | GCAGGGGGCCU | 13-23 |
| SNORA36C | H/ACA | 250 | GGGCAGCUUCC | 21-31 |
| SNORA38B | H/ACA | 217 | CCAGCUUGCUC | 88-98 |
| SNORA84 | H/ACA | 452 | GCUGCCAUAGC | 104-114 |
| SNORD119 | C/D | 21 | GACUGAAGCUGA | 27-38 |
| SNORD121A | C/D | 8 | UAUCUGAUGAUUCCA | 40-54 |
| SNORD121B | C/D | 30 | UUGUAUGUGUG | 42-52 |
| SNORD123 | C/D | 44 | GCGCUGAUUCA | 22-32 |
| SNORD124 | C/D | 55 | CAGAGCUUUGA | 57-67 |
| SNORD125 | C/D | 7 | CUUCCUGAGCACGCU | 29-43 |
| SNORD126 | C/D | 54 | CAGCUGAUCAG | 37-47 |
| SNORD127 | C/D | 1 | CUAAAUGAGGACAACAGUCCCU | 47-68 |
| snR38A | C/D | 32 | GUCUGAAAAUC | 28-38 |
| snR38B | C/D | 42 | CAAAGAAAGGC | 40-50 |
| snR38C | C/D | 19 | UCUUUUGUGAG | 44-54 |
| snR39B | C/D | 35 | UUCGGGACUGA | 23-33 |
| U101 | C/D | 42 | CCUUCACUCCU | 31-41 |
| U102 | C/D | 4 | UGUGAAAAACACAU | 39-52 |
| U103 | C/D | 11 | CCACUUGCCCUCACUGA | 18-34 |
| U103B | C/D | 11 | CCACUUGCCCUCACUGA | 18-34 |
| U104 | C/D | 20 | CUGCUGACGCG | 40-50 |
| U105 | C/D | 291 | GCUGCUGUGAU | 35-45 |
| U105B | C/D | 46 | AGACGCUGUGA | 31-41 |
| U106 | C/D | 134 | UUUCCCCAUCA | 21-31 |
| U107 | H/ACA | 209 | GAGCAGGGGGC | 42726 |
| U108 | H/ACA | 156 | CCUUUCCUGCA | 114-124 |
| U13 | C/D | 248 | GUGCCACCCUU | 56-66 |
| U14A | C/D | 57 | AGUUUCCACCA | 30-40 |
| U14B | C/D | 100 | GUUGGCCAGUU | 59-69 |
| U15A | C/D | 467 | CCCAGGUGGCC | 48-58 |
| U15B | C/D | 649 | CUUGGUCCUUG | 45-55 |
| U16 | C/D | 190 | CUCAGCGACAG | 35-45 |
| U17a | H/ACA | 373 | CCCCGGGCUCU | 31-41 |
| U17b | H/ACA | 676 | CAGCUUCCCAG | 147-157 |
| U18A | C/D | 94 | GGUCCGUGUUU | 26-36 |
| U18B | C/D | 42 | GGUCCGUGUUU | 26-36 |
| U18C | C/D | 34 | UAAGGUCCGUGU | 21-32 |
| U19 | H/ACA | 492 | CCAGGCUCAUG | 20-30 |
| U19-2 | H/ACA | 570 | GUUCUGGUGGC | 100-110 |
| U20 | C/D | 8 | AGAAAAUUCCU | 45-55 |
| U21 | C/D | 147 | GUUGGUCCUUU | 37-47 |
| U22 | C/D | 372 | GGCUGGGGGAG | 76-86 |
| U23 | H/ACA | 488 | CUCCUUUCUGU | 14-24 |
| U24 | C/D | 39 | GAGAUGGUGAUG | 33-44 |
| U25 | C/D | 44 | CUGUACUGAGC | 28-38 |
| U26 | C/D | 82 | UCUCUUUCUGA | 29-39 |
| U27 | C/D | 25 | GCUGAACUUUC | 33-43 |
| U28 | C/D | 34 | UUCUGUGAGGUA | 29-40 |
| U29 | C/D | 8 | GCUCACUAUGA | 21-31 |
| U3 | C/D | 200 | GGGAGUGAGAG | 183-193 |
| U30 | C/D | 85 | GAUGACUUGCU | 34-44 |
| U31 | C/D | 27 | CCAGUCUGAUC | 27-37 |
| U3-2 | C/D | 199 | GGGAGUGAGAG | 183-193 |
| U32A | C/D | 63 | UUUGAGUCUCACG | 31-43 |
| U32B | C/D | 49 | UUUGAGUCUCA | 31-41 |
| U3-2B | C/D | 199 | GGGAGUGAGAG | 183-193 |
| U33 | C/D | 39 | ACAUUCGAGUUUC | 27-39 |
| U3-3 | C/D | 201 | GGGAGUGAGAG | 183-193 |
| U34 | C/D | 8 | GUUUGAUCCUC | 30-40 |
| U3-4 | C/D | 199 | GGGAGUGAGAG | 183-193 |
| U35A | C/D | 308 | GGUCUGCGGAU | 26-36 |
| U35B | C/D | 84 | GCCCACGUGGG | 37-47 |
| U36A | C/D | 12 | UUCAACCUUGA | 27-37 |
| U36B | C/D | 17 | AAAUUACUGUGA | 29-40 |
| U36C | C/D | 7 | CCUGAAUAAACCAU | 28-41 |
| U37 | C/D | 29 | UCACUUUGACC | 23-33 |
| U38A | C/D | 176 | CUGAAGGGAGA | 33-43 |
| U38B | C/D | 144 | UUCUGCUACUG | 25-35 |
| U41 | C/D | 20 | GUUGAUGUGGA | 24-34 |
| U42A | C/D | 1 | GGAAAAGAAUGACAUGAACAAAGG | 21-44 |
| U42B | C/D | 2522 | UGACACUUGUG | 29-39 |
| U43 | C/D | 21 | GGACAGAAACU | 26-36 |
| U44 | C/D | 14 | GAAGGUCUUAA | 34-44 |
| U45A | C/D | 13 | UAUUAUCUGAA | 21-31 |
| U45B | C/D | 18 | UAGCUGAAUCUA | 24-35 |
| U45C | C/D | 9 | UAUUACUACUU | 42-52 |
| U46 | C/D | 446 | GUGGCCGUCUU | 34-44 |
| U47 | C/D | 1 | GUAAAACCGUUCCAUUUUGAU | 36-56 |
| U48 | C/D | 71 | GUCGCUGAUGC | 32-42 |
| U49A | C/D | 8 | AAGUGCCGUCAG | 24-35 |
| U49B | C/D | 9 | UAAUAGGAAGUGCCGUCA | 13-30 |
| U50 | C/D | 9 | CCGAACCUGAACU | 19-31 |
| U50B | C/D | 4 | UCCCGAAGCUGAUAACCU | 19-36 |
| U51 | C/D | 71 | GGCUGAGUUCG | 35-45 |
| U52 | C/D | 46 | CUCCGAUGCUG | 27-37 |
| U53 | C/D | 182 | CUGCUGGCUGA | 28-38 |
| U54 | C/D | 18 | GUACCUAUUGUGUUGAG | 13-29 |
| U55 | C/D | 71 | CGCGGUGGGGA | 41-51 |
| U56 | C/D | 35 | AGUGAGUGUUG | 41-51 |
| U57 | C/D | 38 | CUGACCUUGUA | 23-33 |
| U58A | C/D | 55 | GGACACCUUUG | 20-30 |
| U58B | C/D | 39 | UCUUAGGACACCUUUG | 16-31 |
| U58C | C/D | 13 | GGACACCUUUG | 20-30 |
| U59A | C/D | 97 | UUCUGAGUUUG | 36-46 |
| U59B | C/D | 141 | UCUUCUGAGUU | 34-44 |
| U60 | C/D | 22 | GCUUUGACUUCUGACA | 15-30 |
| U61 | C/D | 12 | CAUUGAUCGUCU | 19-30 |
| U62A | C/D | 106 | ACCCUCCACUG | 32-42 |
| U62B | C/D | 106 | ACCCUCCACUG | 32-42 |
| U63 | C/D | 6 | CUGAAAGAACG | 32-42 |
| U64 | H/ACA | 1429 | GGCCUCUCUUU | 103-113 |
| U65 | H/ACA | 295 | ACUGCACCUGA | 13-23 |
| U66 | H/ACA | 341 | GUGAUGUGGCA | 25-35 |
| U67 | H/ACA | 611 | CUGCCUUCCUU | 112-122 |
| U68 | H/ACA | 394 | GGGGCUGUGGC | 86-96 |
| U69 | H/ACA | 83 | AAGUACUGCCA | 35-45 |
| U70 | H/ACA | 398 | CUCAUGGGGAC | 32-42 |
| U70B | H/ACA | 287 | GGGGGUCCAGU | 36-46 |
| U70C | H/ACA | 399 | UCCUCAUGGGG | 31-41 |
| U70D | H/ACA | 314 | UUCCUCAGGGA | 29-39 |
| U70E | H/ACA | 838 | CCUCUUUGGGG | 31-41 |
| U70F | H/ACA | 338 | AUGGGGGCCCA | 35-45 |
| U70G | H/ACA | 333 | CAUGGGGGGGC | 34-44 |
| U71a | H/ACA | 232 | UUGGAGGGUCC | 89-99 |
| U71b | H/ACA | 285 | CCUUUGCCCUG | 29-39 |
| U71c | H/ACA | 379 | UGCCCUGGUCA | 33-43 |
| U71d | H/ACA | 412 | CUGCCUGUGCCC | 26-37 |
| U72 | H/ACA | 323 | CUAGUUCUUUC | 87-97 |
| U73a | C/D | 43 | CAGAUGAUGGC | 27-37 |
| U73b | C/D | 9 | CCCAAAUGAUACAUACU | 26-42 |
| U74 | C/D | 66 | UGGUAGGGACA | 22-32 |
| U75 | C/D | 41 | GUGGACAGAAG | 22-32 |
| U76 | C/D | 79 | GAGUGCUAGAA | 32-42 |
| U77 | C/D | 23 | GUUCAGCAGAU | 21-31 |
| U78 | C/D | 27 | CCUGAAAUGAG | 27-37 |
| U79 | C/D | 40 | AAUCUCUCUGA | 37-47 |
| U8 | C/D | 5 | CUUGCAACACCCUGAUUGCUCC | 105-126 |
| U80 | C/D | 17 | UCAGCAGACUA | 22-32 |
| U81 | C/D | 28 | GAACUCUCUCA | 29-39 |
| U82 | C/D | 5 | ACCUGAUGUUA | 40-50 |
| U83 | C/D | 16 | GUGCAAGGACG | 35-45 |
| U83A | C/D | 114 | UGAGCGCUGGG | 24-34 |
| U83B | C/D | 75 | UGCGCUGGGCAC | 26-37 |
| U84 | C/D | 71 | GCAGUGAUGAC | 39-49 |
| U86 | C/D | 222 | GGGGGUGAUGG | 52-62 |
| U94 | C/D | 286 | GUGGCAGGCUG | 83-93 |
| U95 | C/D | 57 | UGUCGGUGCUG | 29-39 |
| U96a | C/D | 117 | GGGAGUGAGGA | 40-50 |
| U96b | C/D | 76 | GGUAGUGAGGA | 40-50 |
| U97 | C/D | 103 | GGAGUUCUUGA | 47-57 |
| U98b | H/ACA | 209 | CUUCCAUGUAG | 24-34 |
| U99 | H/ACA | 445 | UGGCGGCUUCC | 102-112 |
| Z17B | C/D | 34 | CCUGAUCUUUG | 33-43 |
