# Supplementary material for: Enhanced snoMEN Vectors Facilitate Establishment of GFP–HIF-1α Protein Replacement Human Cell Lines
Source: PLoS One. 2016 Apr 29;11(4):e0154759. doi: 10.1371/journal.pone.0154759 (PMC4851398; doi:10.1371/journal.pone.0154759)
Supplement: S1 File — (PDF) [file pone.0154759.s003.pdf]

Supporting Information

Supporting information Captions

S1 File. Supporting information of the study "Analysis of the enhanced snoMEN vector: An efficient way of protein replacement in human cells". Northern and high sensitivity RNA blot analysis **(Supplementary Methods).** Potential mRNA targets of snoRNAs (**Figure A**). Subcellular localisation of box H/ACA snoRNA-like miRNA miR-566 precursor (**Figure B**). The vector structure for targeted suppression of GFP fusion proteins (**Figures C-E**). Comparison of targeted suppression of GFP fusion proteins using snoMEN vectors (**Figure F**).

Supplementary Methods

Northern and High sensitivity RNA blot analysis. HeLa cell extracts were fractionated using sucrose gradients, as previously described [7, 33]. Total HeLa cell RNA and RNA from separate cytoplasmic, nucleoplasmic and nucleolar fractions were isolated using the TRIzol method, with DNase I treatment, according to the manufacturer's instruction (Invitrogen). Equal amounts of RNA (100 µg) from each sample were separated by 8M Urea polyacrylamide denaturing gel electrophoresis in 1xTBE buffer and the RNA transferred onto nylon membrane (Hybond-N; Amersham) by electro blotting. After either UV crosslinking, or chemical cross linking, for high sensitivity RNA blot, the membrane was hybridized with ^{32}P 5' end-labelled oligoribonucleotide probes specific for the following RNA species; (miR-566: 5'-GUUGGGAUCACAGGCGCCC-3', HBII-180C: 5'-GUGCACUGUGUCCUCAGGGGUG-3', tRNA-Ile 5'-UGGUGGCCCGUACGGGGAUCGA-3'). High sensitivity RNA blots were prepared as previously described[45].

A Fig. Potential mRNA targets of snoRNAs. Two predicted complementary target gene sequences with highest complementarity are shown for each snoRNA (ARHGAP24: Rho GTPase activating protein 24 isoform 1, CDKAL1: CDK5 regulatory subunit associated protein 1-like 1, FMN1: Formin-1, RGS22: regulator of G-protein signaling 22, CADM3: cell adhesion molecule 3, CPNE5: copine-5, PCYOX1: prenylcysteine oxidase 1, FBXO22: F-box protein 22).

B Fig. Subcellular localisation of box H/ACA snoRNA-like miRNA miR-566 precursor. Northern blot of HeLa cell extracts fractionated into cytoplasmic (lane 2), nucleoplasmic (lane 3) and nucleolar (lane 4) fractions were probed for the presence of miR-566, HBII-180C and tRNA-Ile encoding molecules. In all panels, bands labeled with 'pri-miR-566' represent the expected size of the predicted snoRNA, those labeled with 'pre-miR-566' represent the expected size for the miRNA hairpins and 'miR-566' represents the expected size of the mature miRNA/sdRNA. The bands indicated by arrowhead were not always observed. Blot was confirmed by two independent experiments.

C-E Figs. The vector structure of a targeted suppression of GFP fusion proteins. This shows the same information as in **Fig 1b** except the snoRNA/snoMEN are U77 (**C**), ACA16 (**D**) and miR-566 (**E**). The resulting snoMEN were subcloned into the 5' region of the vector with mCherry fluorescent protein cDNA. Diagram shows complementary regions of snoMEN targeting GFP cDNA sequence (Anti-GFP mRNA). A mutant snoMEN expression plasmid with the box D/box H/ACA core motif mutated was also constructed, shown as BoxDmut/BoxACAmut on the sequence.

F Fig. Comparison of targeted suppression of GFP fusion proteins using snoMEN vectors. This shows the same experiment as in **Fig 4a** except transfected plasmids encode ACA16snoMEN and miR-566snoMEN. The effect of ACA16 snoMEN (left panel) and miR-566 snoMEN (right panel) constructs on GFP-SMN1 expression in the $HeLa^{GFP\text{-}SMN}$ stable cell line, which expresses GFP fused at the amino terminus of SMN1. Images show the effect of transfecting either wild type snoRNAs, encoded in mCherry expression plasmid mCherry-N1 (WT, Control), expression plasmid ACA16/miR-566 snoMEN (ACA16snoMEN at left panel, miR-566snoMEN at right panel), or expression plasmid of ACA16/miR-566 boxACA mutant snoMEN (ACA16snoMEN box ACAmut at left panel, miR-566snoMEN box ACAmut at right panel), in the $HeLa^{GFP\text{-}SMN}$ stable cell lines. Upper panels show GFP and mCherry fluorescence signals of images recorded from fixed cells (GFP and mCherry). Lower panels show merged images combining the GFP (green) and mCherry (red) signals. Scale bar is 10 μm. The arrows indicate transfected cells and arrowheads indicate cells showing reduced GFP signals.

Fig A

U47 box C/D snoRNA

ARHGAP24

```
5' AUAUAAUGAUAUCACUGUAAAAC 3'
   ||||||||||||| |||||||||
3' UAUAUUACUAUAGCGACAUUUUG 5'
```

CDKAL1

```
5' AUAUAAUGAUAUCACUGUAAAAC 3'
   ||||||||||| || |||||||
3' UAUAUUACUAUCGUAACAUUUUA 5'
```

U77 box C/D snoRNA

FMN1

```
5' UUGCAUAGUUCAGCAGAUUUAAU 3'
   ||||||||||||||||||| |||
3' AACGUAUCAAGUCGUCUAACUUA 5'
```

RGS22

```
5' UUGCAUAGUUCAGCAGAUUUAAU 3'
   ||||||||||||||||||| |||
3' AACGUAUCAAGUCGUCUAACUUA 5'
```

ACA16 boxH/ACA snoRNA

CADM3

```
5' GCCCAGAGUGACAGUUUUCCUUGACGGU 3'
   || |||||||||||||||| ||||| ||
3' CGAGUCUCACUGUCAAAAGAAACUGUCA 5'
```

CPNE5

```
5' GCCCAGAGUGACAGUUUUCCUUGACGGU 3'
   |  ||||||| |||| |||||||| ||
3' C-AGTCTCACAGTCAGAAGGAACTACCC 5'
```

miR-566 precursor (H/ACA like)

PCYOX1

```
5' GGGCGCCUGUGAUCCCAACUAC 3'
   ||||||||||||||||||||||
3' CCCGCGGACACUAGGGUUGAUG 5'
```

FBXO22

```
5' GGGCGCCUGUGAUCCCAACUAC 3'
   ||||||||||||||||| ||||
3' CCCGCGGACACUAGGGUCGATG 5'
```

Fig B

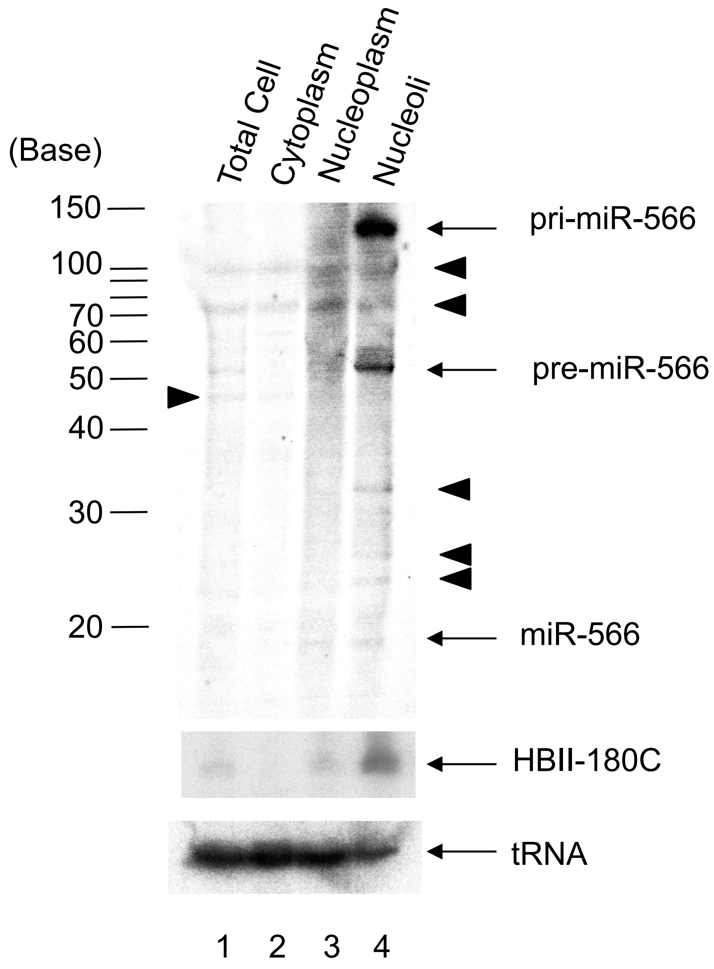

(Base)
Total Cell
Cytoplasm
Nucleoplasm
Nucleoli
150
100
70
60
50
40
30
20
pri-miR-566
pre-miR-566
miR-566
HBII-180C
tRNA
1 2 3 4

Fig C

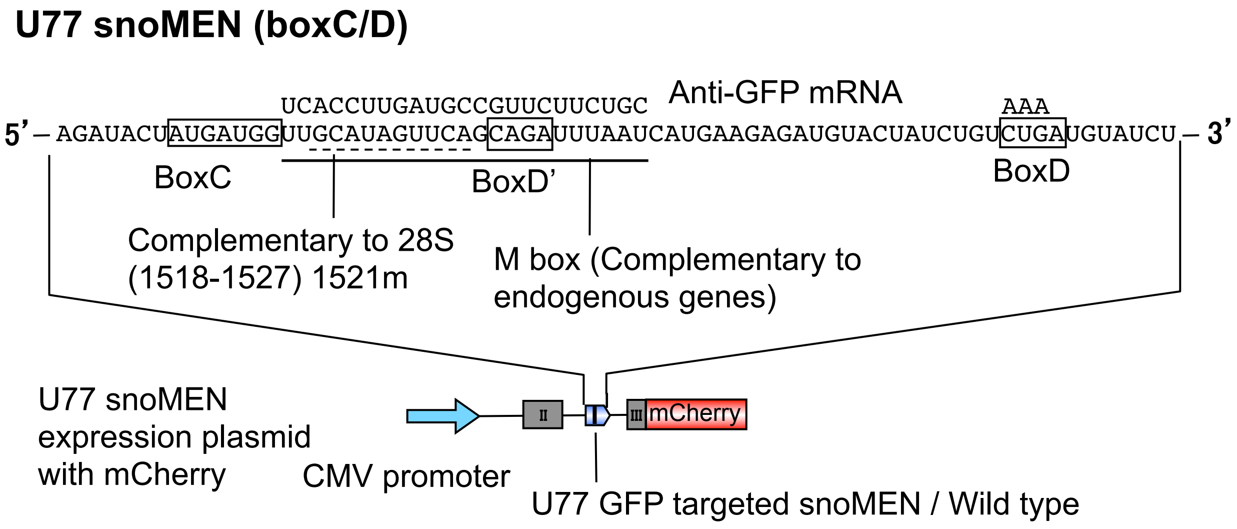

U77 snoMEN (boxC/D)
UCACCUUGAUGCCGUUCUUCUGC
Anti-GFP mRNA
AAA
5'– AGAUACUAUGAUGGUUGCAUAGUUCAGCAGAUUUAAUCAUGAAGAGAUGUACUAUCUGUCUGAUGUAUCU – 3'
BoxC
BoxD'
BoxD
Complementary to 28S (1518-1527) 1521m
M box (Complementary to endogenous genes)
U77 snoMEN expression plasmid with mCherry
CMV promoter
II
III
mCherry
U77 GFP targeted snoMEN / Wild type

Fig D

ACA16 snoMEN (boxH/ACA)

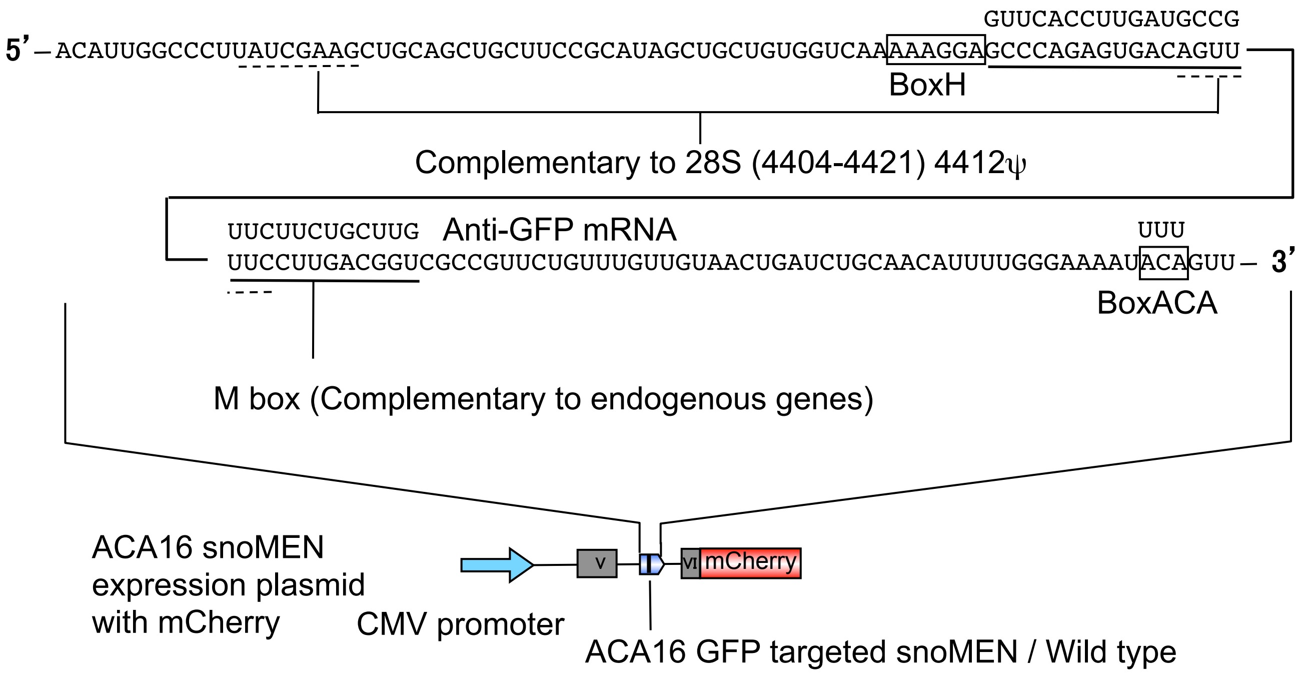

Fig E

miR-566 snoMEN (boxH/ACA)

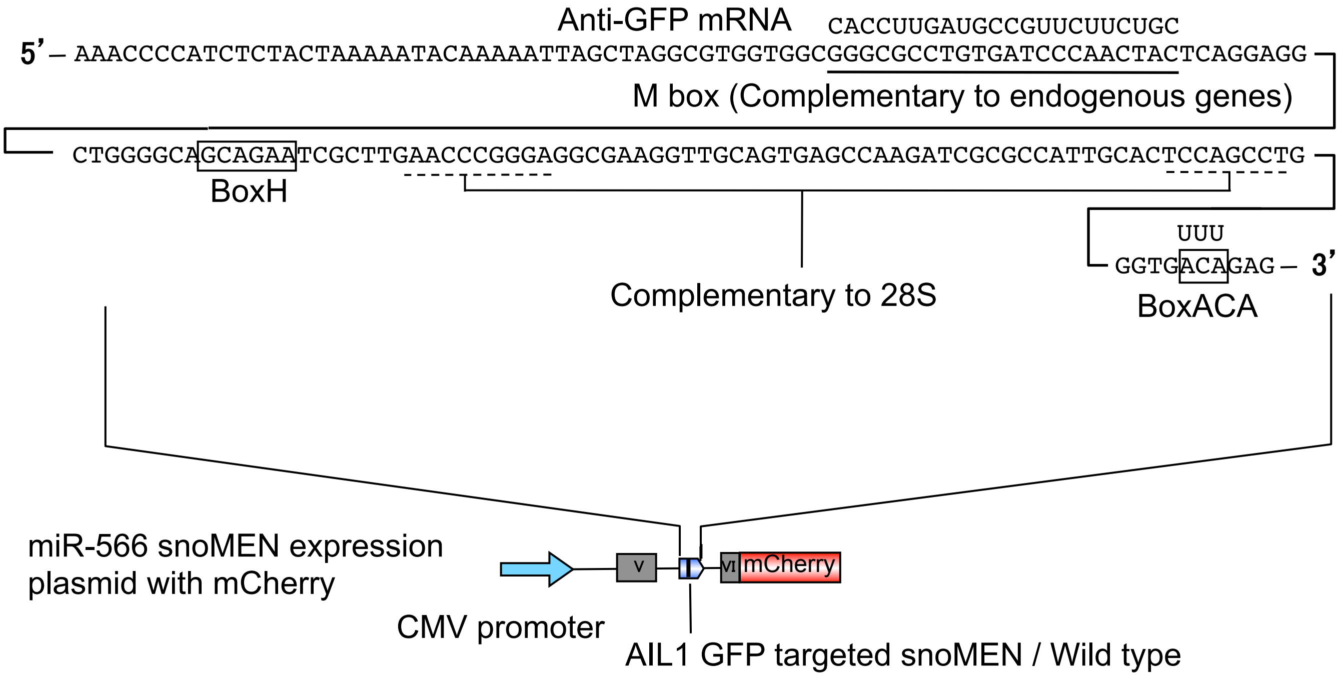

Fig F

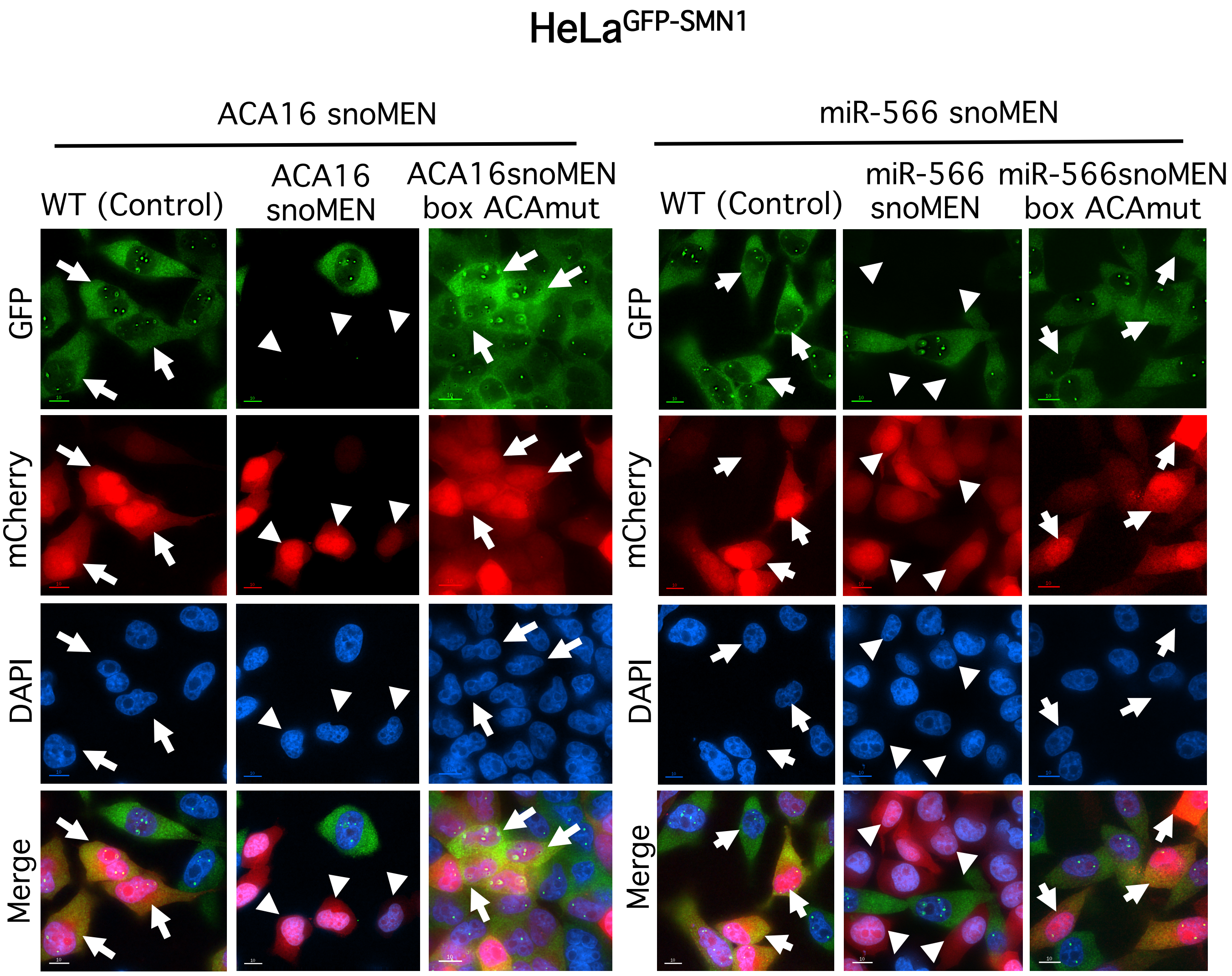

HeLa$^{GFP-SMN1}$
ACA16 snoMEN
miR-566 snoMEN
WT (Control)
ACA16 snoMEN
ACA16snoMEN box ACAmut
WT (Control)
miR-566 snoMEN
miR-566snoMEN box ACAmut
GFP
mCherry
DAPI
Merge
GFP
mCherry
DAPI
Merge
